# Investigating “Another Me” digital twin technology to support the development of human relationships: An exploratory randomized controlled study

**DOI:** 10.1371/journal.pone.0353835

**Published:** 2026-08-07

**Authors:** Ami Yamasato, Chihiro Takayama, Shinichiro Eitoku, Yoko Ishii, Ryo Ishii, Lidwina Andarini, Kazuya Matsuo, Atsushi Otsuka, Setsukou Sha, Jun Tamura, Emi Kamono, Mizuki Ohashi, Yuichiro Yano, Tomoyuki Miyazaki

**Affiliations:** 1 Center for Promotion of Research and Industry-Academic Collaboration, Yokohama City University, Yokohama, Kanagawa, Japan; 2 Human Informatics Laboratories, NTT, Inc., Yokosuka, Kanagawa, Japan; 3 Department of Biostatistics, Graduate School of Medicine, Yokohama City University, Yokohama, Kanagawa, Japan; 4 Department of General Medicine, Faculty of Medicine, Juntendo University, Tokyo, Japan; Japanese Red Cross Medical Center, JAPAN

## Abstract

Social anxiety disorder has a considerable negative impact on educational attainment, employment status, and relationship development. Exposure therapy is an established method used to treat social anxiety by getting patients used to talking to other people. However, this procedure requires patients to have conversations with strangers, which may impose a psychological burden. This study aimed to examine whether the burden could be reduced during a first-time face-to-face interaction by using a human digital twin avatar technology that we call “Another Me.” We conducted an exploratory, randomized, open-label, controlled study with 30 young participants who self-reported nervousness during conversations with strangers. Participants were divided into an intervention group and a control group. The intervention group (n = 15) watched a generated video of a conversation between their Another Me and the Another Me of an assigned interlocutor. After viewing the video, they engaged in an online conversation with the assigned interlocutor. The control group (n = 15) watched a video of a conversation between the avatars of two strangers and then engaged in a conversation with an assigned interlocutor. Results showed that the primary endpoint—the between-group difference in heart rate change from before to after the online conversation—was not statistically significant. However, exploratory secondary analyses suggested that heart rate decreased in the intervention group after viewing the pre-dialogue simulation video and remained relatively stable until the completion of the online conversation. In contrast, the control group exhibited an increase in heart rate before the online conversation. Additionally, the intervention group reported lower anxiety levels and a greater willingness to converse until the end of the face-to-face conversation. These findings should be interpreted as preliminary and hypothesis-generating. Further confirmatory trials with rigorous physiological monitoring are required.

## Introduction

Social anxiety refers to a persistent fear of social situations and interactions. Individuals with social anxiety often experience anxiety in situations such as speaking in public, interactions with strangers, and attending parties [1]. These fears can cause substantial distress and impairment in educational attainment, employment status, and relationship building and are associated with impaired social functioning, social isolation, and even suicidal thoughts [2–4]. A traditional treatment for social anxiety is exposure therapy, where patients are encouraged to repeatedly expose themselves to stressful situations in which they are forced to interact with others, progressing from less fearful to more challenging situations as they gain confidence [5–7]. However, the psychological burden of exposure to strangers often deters patients from continuing the therapy [8]. Furthermore, psychotherapists must spend a great deal of time and effort identifying subtle patterns of avoidance and encouraging patients to engage in the opposite behavior, which imposes a financial burden on the patients [6,9].

Exposure therapy with video feedback can alleviate these problems. Causes of social anxiety include distorted and negative self-image, egocentric attention, and the negative effects of safety behaviors (e.g., influencing the behavior of others), all of which contribute to the persistence of social anxiety. Video feedback is particularly effective in repairing distorted self-images. Warnock-Parkes et al. [10] examined the effects of video feedback on patients with social anxiety. In their study, participants conversed with strangers and viewed a video recording of the interaction. Before viewing the video, they predicted how it would appear, and after viewing the video, they made a second self-assessment. Consequently, 98% of the patients improved their self-assessments after receiving video feedback, and one week after the video feedback, there was a significant decrease in social anxiety (as measured using the Liebowitz Social Anxiety Scale). Other studies have reported the effectiveness of video feedback on social anxiety in clinical and nonclinical settings [11–15]. Video feedback is a key component of cognitive therapy for social anxiety as it helps modify negative self-perceptions and gain insight into the effects of safety behaviors. Video feedback methods based on cognitive behavioral therapy support image formation by encouraging the correction of negative self-images and promoting the internalization of successful experiences through video self-monitoring [16–19]. However, exposure therapy using video feedback has not eliminated the excessive psychological burden of exposure to others, and there are still retention challenges [8].

In the current study, our objective is to reduce the psychological burden by using a novel video feedback method in which a participant’s digital twin (a digital representation reflecting individual characteristics), rather than the participants themselves, is exposed to a stranger. Our approach utilizes digital twin technology called “Another Me” to reproduce a participant’s appearance, voice, and knowledge in the form of an avatar that communicates with a stranger smoothly in a digital space. Our hope is that viewing their own Another Me videos will correct participants’ negative self-image and help them feel it is easy to imagine a successful communication experience with a stranger, thereby reducing social anxiety.

We conducted an exploratory, randomized, open-label, controlled study with 30 young participants who reported feeling nervous during conversations with strangers. The intervention group (n = 15) participated in an online one-on-one conversation after viewing a video of their Another Me engaging in a conversation with an interlocutor avatar. In contrast, the control group (n = 15) participated after viewing a video showing a conversation between two strangers. Our aim is to determine whether there is an alleviating effect of exposure therapy with video feedback using Another Me on anxiety and tension that is experienced in subsequent face-to-face conversations.

## Materials and methods

### Study design

This study was a randomized, open-label, controlled trial targeting young adults who self-reported experiencing nervousness when conversing with unfamiliar people. Allocation was performed using the minimization method, with sex (male or female) as the allocation factor, and participants were assigned to the intervention and control groups in a 1:1 ratio.

For study participants who provided written informed consent, we prepared a case registration form, with their personal information removed, and emailed it to the registration center. The registration center determined eligibility based on the information in the case registration form, and if a participant was determined to be eligible, the allocation was conducted. Allocation was performed by a contract research organization (Nouvelle Plus Inc.) per the CONSORT statement. All participants in this study viewed the Another Me video after randomization, and data on the primary endpoint were included in the full analysis set (FAS).

Initially, we planned to measure the continuous heart rate during the experiment using smartwatches (vívosmart 5: GARMIN) as the primary endpoint. However, due to a system malfunction in the data extraction application, the endpoint had to be changed midway through the study period. Upon making the change, an amendment application was immediately submitted to the Ethics Committee, and after obtaining approval, the endpoint was changed to heart rate measured using the upper-arm blood pressure monitor (HCR-7201: OMRON Corp.). This change was implemented after all data collection had been completed and before data fixation and the initiation of statistical analyses.

### Participants

The criteria for participant selection were (1) adults aged 18 years or older, (2) self-reported nervousness when speaking to new people, (3) no difficulty in speaking Japanese, (4) no history of atrial fibrillation or arrhythmia, and (5) provision of written consent to participate in the study.

Recruitment occurred from February 14 to April 3, 2024, and participants were followed until their final outcome assessment on April 12, 2024. The study was publicized and announcements were made during lectures at Yokohama City University, on the university’s portal site, and through the business chat tool of the research center.

### Interventions

We conducted a comparison between the two groups to assess the effect of viewing dialogue videos (Another Me videos) featuring avatars (digital twins; Details of the avatar creation process are provided in S1 Fig) that replicated themselves. The intervention group viewed dialogues between the digital twins that reproduced themselves and their assigned interlocutors, and the control group viewed dialogues between the digital twins of two strangers. Another Me videos used in this experiment were generated using the method described in based on photos of the participants’ face, a few seconds of speech audio, and profile information. Details of the video generation process are provided in S2 Fig (for further details, refer to Appendix S1 File) [20]. The experiment was conducted entirely online, with participants accessing the survey response site and conferencing system from home, and it took approximately 60 min to complete. All the participants and experimenters were meeting for the first time.

The experiment comprised three sections: (1) preparation, (2) viewing an Another Me video, and (3) online conversation.

(1) Preparation (before viewing the videos)

The participants accessed the experimental site at a designated time and completed a pre-questionnaire regarding the psychological data items described in the Psychological Data section. To enhance participants’ sense of self-identification with their avatars, only participants in the intervention group were asked to select the avatar they felt most resembled them. The options included an avatar created directly from the participant’s photo and two avatars created from photos that were a 50% morphing of the participant and two experimenters. Subsequently, both the intervention and control groups wore blood pressure monitors and had their blood pressure measured.

(2) Viewing an Another Me video

Participants in the intervention group viewed Another Me videos featuring the avatar they selected in step (1) and the avatar of the person they would interact with online in step (3). Participants in the control group viewed Another Me videos featuring two experimenters. After viewing, all participants completed a post-viewing questionnaire and had their blood pressure measured.

(3) Online conversation

After preparing for an online conversation using the Zoom communication system, the participants completed a questionnaire and had their blood pressure measured. Afterward, they had a one-on-one online conversation with an assigned interlocutor. No specific topic was assigned, and the participants talked freely for approximately 10 minutes. Participants in the intervention group interacted with the experimenter, who appeared in the video they had viewed in advance. In contrast, participants in the control group interacted with an experimenter who was not represented as a digital twin in the video. After the conversation, all participants completed a post-dialogue questionnaire and had their blood pressure measured.

### Measurements and biometric data

Biometric data, including heart rate and blood pressure, were measured using an upper-arm blood pressure monitor (HCR-7201; OMRON Corp.). The measurable range of pulse rate was 40–180 beats per minute. Blood pressure was measured at two levels: when the heart contracted (systolic blood pressure) and when the heart expanded (diastolic blood pressure). The heart rate and blood pressure were measured at four points: before and after viewing the video and before and after the online conversation.

### Psychological data

The following scales were measured via online questionnaires: Liebowitz Social Anxiety Scale Japanese version (LSAS-J) [21,22], Shyness Scale Japanese version (Social Reticence Scale) [23], Mood Inventory [24], and Subjective Units of Distress Scale (SUDs) [25]. Additionally, items related to tension based on SUDs and items measuring willingness to talk were included.

The LSAS-J was used to assess the severity of social anxiety disorder. It consists of 24 items that ask about fear and avoidance behaviors in various social situations. Respondents answered each item on a four-point scale, indicating how much it applies to them. The Japanese version was developed to reflect Japanese culture and its social background. Scores are divided into five categories: “Not social anxiety (0–29),” “Mild social anxiety (30–49),” “Moderate social anxiety (50–69),” “Marked social anxiety (70–89),” and “Very severe social anxiety (90 or above).” The LSAS-J was administered once before viewing the video.

The Japanese version of the Shyness Scale assesses shyness and embarrassment. This scale consists of 23 items that assess reactions and feelings in social situations and interpersonal relationships. Respondents answered each item on a five-point scale, indicating how much it applies to them. The Japanese version has been translated and adjusted to reflect Japanese culture and language. The Shyness Scale was utilized once before viewing the video.

The Mood Inventory assesses an individual’s mood and emotional state. This scale comprises 40 items that measure various emotions and sensations. Respondents were asked to evaluate their current mood and recent emotional state. They answered each item on a four-point scale, indicating how much it applies to them. The Mood Inventory was administered at three points: before viewing the video and before and after the online conversation.

Regarding tension, participants used a questionnaire based on SUDs to rate their current state on an 11-point scale, with 10 indicating the highest level of tension and 0 indicating no tension. Anxiety and tension were measured at four points: before and after viewing the video and before and after the online conversation.

Regarding willingness to converse, participants were asked, “How willing are you to converse?” and rated their willingness on an 11-point scale ranging from “0: Not at all willing” to “10: Very willing.” Measurements were taken before viewing the video and before the online conversation. Before viewing the video, participants were asked about their “willingness to converse in everyday life,” and before the online conversation, they were asked about their “willingness to converse in the upcoming conversation.”

To assess how strongly participants perceived the avatar appearing in the viewed video as resembling themselves, we administered a Japanese-translated version of the Avatar Identification/Perceived Similarity subscale from the Player Identification Scale [26]. Both groups of participants rated the extent to which the avatar in the viewed video felt similar to themselves. This measure was administered after video viewing. As a validated Japanese version of this scale is not currently available, the translated items were used for exploratory purposes.

### Feasibility assessment

The feasibility of the intervention was assessed based on the following criteria: (1) study completion rate, calculated as the percentage of enrolled participants who completed all scheduled evaluations, and (2) occurrence of adverse events or side effects during the intervention, monitored through participant self-reporting.

### Statistical analysis

Based on previous studies, we assumed that the mean change in heart rate in each group would decrease by at least 2.6 beats/min before and after the conversation, with a standard deviation of 5 beats/min for the change [27]. To observe the reduction in heart rate within each group, we set the sample size to 15 participants per group, expecting a 95% confidence interval width of 5.2 beats/min. The sample size was set to 32, assuming a dropout rate of 5%. The analysis was performed using the full analysis set, and all participants who received the intervention were grouped according to their allocations.

The primary endpoint of this study was the change in heart rate before and after the online conversation. In the main analysis, the between-group difference in heart rate change was evaluated using analysis of convariance (ANCOVA), with sex included as a covariate. We also calculated the 95% confidence interval for the between-group difference. In addition, 95% confidence intervals were calculated for the within-group changes in heart rate before and after the online conversation. As secondary analyses, we descriptively summarized heart rate at four time points (before and after video viewing and before and after the online conversation) to examine time-course patterns.

The secondary endpoints were blood pressure, SUDs (anxiety and tension), mood questionnaires, and motivation to talk. These endpoints were analyzed descriptively by calculating means and 95% confidence intervals without formal hypothesis testing. Accordingly, these analyses were considered descriptive and hypothesis-generating. The confidence level was set at 95%, and the significance threshold at *p* < 0.05. All statistical analyses were performed using the R software, version 4.4.0 (R Foundation for Statistical Computing, Vienna, Austria).

### Ethical considerations

This study was approved by Yokohama City University’s “Ethics Committee for Life Science and Medical Research Involving Human Subjects” (approval date: February 14, 2024, approval number: General 2023−035).

Using an explanation and consent form approved by the ethics review committee, we provided an in-person explanation to the participants and obtained their written consent. We also explained that they could withdraw their consent at any time during the study period and would not suffer any disadvantages in refusing to participate or withdrawing their consent.

The individuals shown in this S1 Figs. are co-authors and have given written informed consent (as outlined in the PLOS consent form) to publish these images.

The research summary and results of this study have been registered in the public database (University Hospital Medical Information Network: UMIN-CTR) established by the National University Hospital Directors’ Association and others, and the information will be updated appropriately as the research progresses (registration date: February 15, 2024; registration number: UMIN000053617).

### Reporting guideline

We used the CONSORT 2025 reporting guideline to draft this manuscript [28], and the CONSORT reporting checklist when editing [29], included in Supplement 2.

## Results

**Fig 1:**
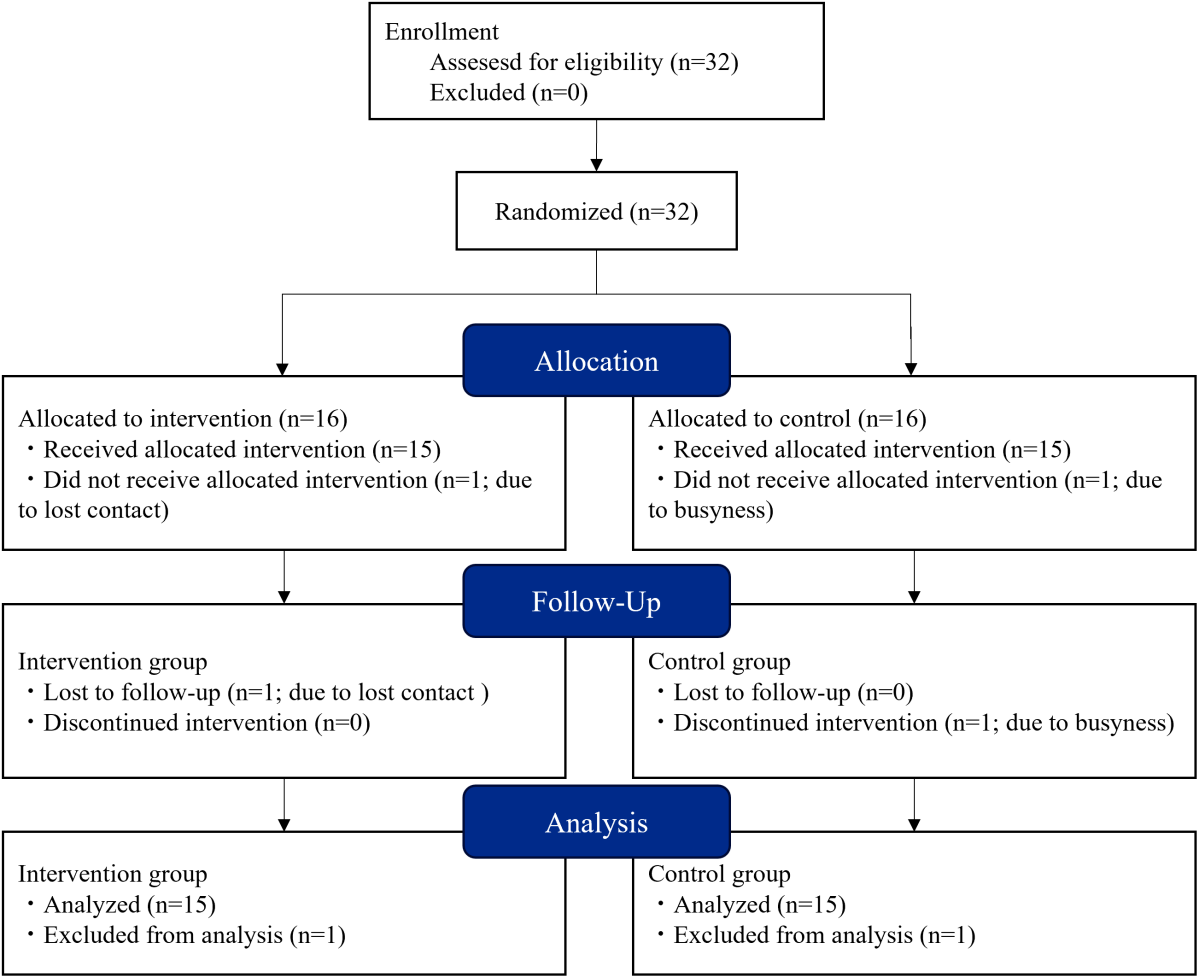
Flowchart of the study process. Flowchart illustrating the study procedure from obtaining informed consent to data analysis.

Fig 1 shows the study flow, which starts with recruiting the study participants for the analysis. A total of 32 participants were individually informed about the study, and consent was obtained from all of them. Two participants (one in the intervention group and the other in the control group) dropped out (dropout rate: 6.25%), and 30 participants completed the study (completion rate: 93.8%). As for the reasons for the dropout, one participant in the control group declined to participate in the study due to being busy (cessation before the start of the intervention), and one participant in the intervention group could not be followed up (cessation before the intervention). Although background information for these two participants was obtained, psychological scales and biometric indicators were not assessed.

### Baseline characteristics

The 30 participants were aged 19–24 years (mean = 20.9, SD = 1.51), and there were seven males (23%) and 23 females (77%). None of the participants in either group had LSAS anxiety scores in the normal range (*LSAS_anxiety*), and the largest proportion of participants fell into the moderate range. The avoidance scores (*LSAS_avoidance*) were distributed in the normal-to-moderate range, indicating that both groups had similar levels of social anxiety. The Shyness Scale showed high values for both groups, with 79.5 ± 13.90 for the intervention group and 72.6 ± 14.79 for the control group (Table 1).

**Table 1:** Baseline characteristics.

Group	Intervention group	Control group
N	15	15
*Age* (y)		
mean(sd)	21.3(1.71)	20.6(1.24)
median(IQR)	21(20, 22)	20(20, 21)
(min,max)	(19, 24)	(19, 24)
*Sex* (%)		
Female	11(73.3%)	12(80.0%)
Male	4(26.7%)	3(20.0%)
LSAS unit Frequency (%)		
*LSAS_anxiety*		
Not social anxiety (0–29)	0(0%)	0(0%)
Mild social anxiety (30–49)	5(33.3%)	4(26.7%)
Moderate social anxiety (50–69)	7(46.7%)	9(60.0%)
Marked social anxiety (70–89)	2(13.3%)	2(13.3%)
Very severe social anxiety (90-)	1(6.7%)	0(0%)
*LSAS_avoidance*		
Not social anxiety (0–29)	7(46.7%)	6(40.0%)
Mild social anxiety (30–49)	7(46.7%)	8(53.3%)
Moderate social anxiety (50–69)	1(6.7%)	1(6.7%)
Marked social anxiety (70–89)	0(0%)	0(0%)
Very severe social anxiety (90-)	0(0%)	0(0%)
*Shyness*		
mean(sd)	79.5(13.90)	72.6(14.79)
median(IQR)	77(70.5, 91.5)	75(63.5, 85.0)
(min, max)	(52, 100)	(39, 92)

### Primary endpoint

**Fig 2:**
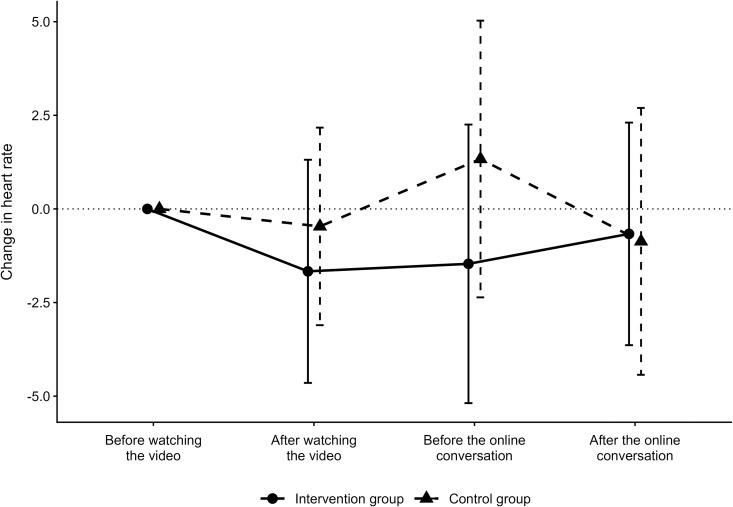
Changes in heart rate over time. Changes in heart rate measured at four time points: before and after video viewing, and before and after the online conversation.

The changes in heart rate between before and after the online conversation for each group were +0.8 ± 7.87 in the intervention group and –2.2 ± 5.29 in the control group (Table 2). Secondary analyses were conducted to further explore heart rate changes at different time points (Fig 2). The changes in heart rate between before and after video viewing were –0.5 ± 4.76 beats/min in the control group and –1.7 ± 5.38 beats/min in the intervention group, indicating a greater decrease in the intervention group. Furthermore, the changes in heart rate between before video viewing and before the online conversation were +1.3 ± 6.67 beats/min in control group, whereas it was –1.5 ± 0.72 beats/min in the intervention group.

**Table 2:** Comparison of changes in heart rate before and after online conversation.

Group	Intervention group	Control group	*p* value	95%CI
N	15	15		
mean (sd)	0.8 (7.87)	−2.2 (5.29)	0.269	[-7.9, 2.3]
median (IQR)	0 (−4,4)	−1 (−5.5, 1.5)		
(min, max)	(−14, 20)	(−11, 6)		

### Secondary endpoints

**Fig 3:**
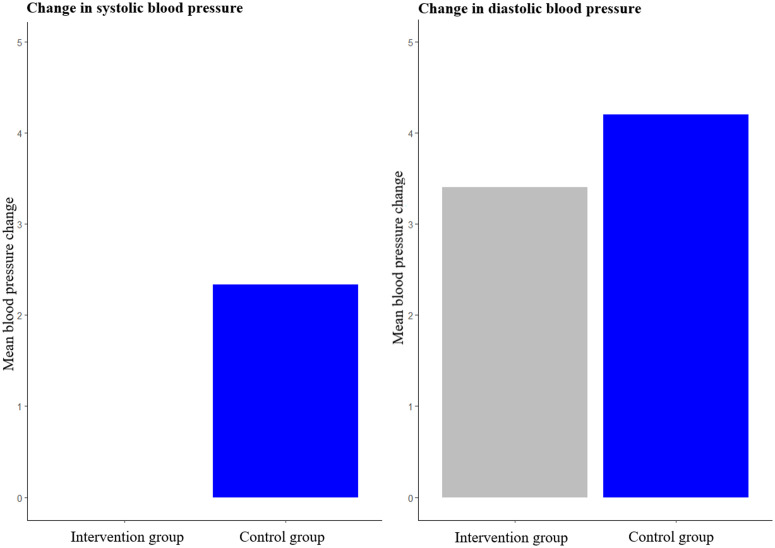
Changes in blood pressure after the online conversation. Changes in systolic and diastolic blood pressure after the online conversation in each group.

**Fig 4:**
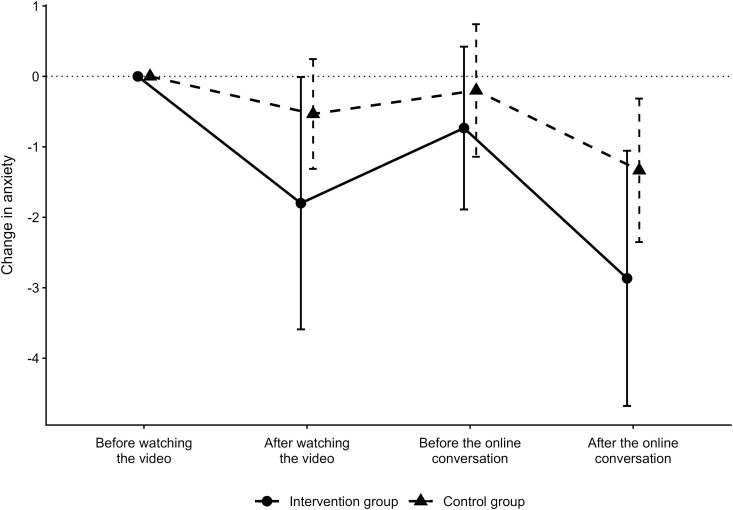
Changes in anxiety over time. Changes in anxiety levels assessed using the Subjective Units of Distress Scale (SUDs) at four time points: before and after video viewing, and before and after the online conversation.

**Fig 5:**
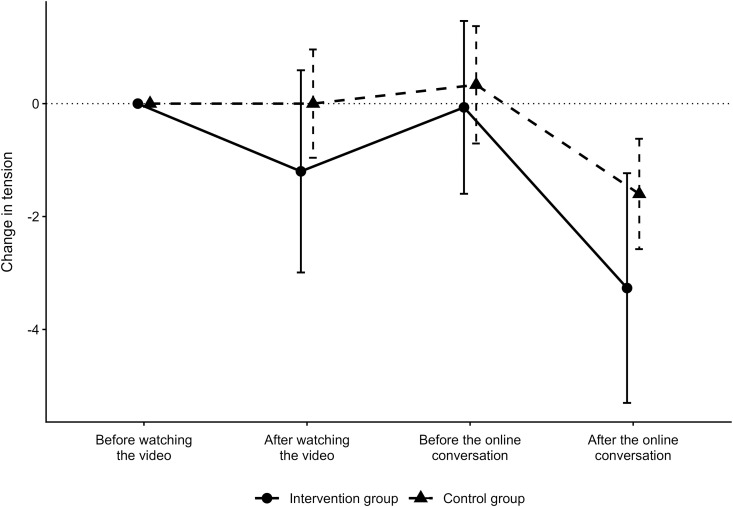
Changes in tension over time. Changes in tension levels assessed using the Subjective Units of Distress Scale (SUDs) at four time points: before and after video viewing, and before and after the online conversation.

Table 3 shows the changes in blood pressure, SUDs, and Mood Inventory scores for each group before and after the online conversations. After the online conversation, systolic blood pressure changed by 0.0 ± 8.59 mmHg in the intervention group and +2.3 ± 5.97 mmHg in the control group. Diastolic blood pressure changed by +3.4 ± 5.12 mmHg in the intervention group and +4.2 ± 7.43 mmHg in the control group (Fig 3). The changes in anxiety before and after the online conversation were –2.1 ± 2.97 in the intervention group and –1.1 ± 1.64 in the control group. Similarly, the changes in tension were –3.2 ± 3.26 in the intervention group and –1.9 ± 1.39 in the control group. Across the four measurement time points, the intervention group showed a change of –1.8 ± 3.23 in anxiety and –1.2 ± 3.23 in tension after viewing the video. In contrast, the control group showed a smaller change in anxiety (–0.5 ± 1.41) and no change in tension (0 ± 1.73) after viewing the video (Figs 4 and 5).

**Table 3:** Comparison of secondary outcomes before and after online conversation.

Group	Intervention group	Control group	Difference between the two groups
N	15	15	
*Blood pressure*	M (SD)	M (SD)	95% CI
Systolic blood pressure	0 (8.59)	2.3 (5.97)	[-2.82, 7.41]
Diastolic blood pressure	3.4 (5.12)	4.2 (7.43)	[-3.37, 5.85]
*SUDs*			
Questionnaire on Anxiety	−2.1 (2.97)	−1.1 (1.64)	[-0.80, 2.65]
Questionnaire on tension	−3.2 (3.26)	−1.9 (1.39)	[-0.61, 2.43]
*Mood Inventory*			
Total	−6.5 (9.96)	−7.5 (8.87)	[-8.22, 5.61]
Tension and excitement	−1.7 (3.29)	−2.3 (2.52)	[-2.77, 1.75]
Refreshing mood	1.7 (3.99)	1 (2.65)	[-3.13, 1.78]
Fatigue	−1.7 (2.96)	−1.6 (2.75)	[-2.66, 1.55]
Depressive mood	−2.1 (3.86)	−1.9 (2.07)	[-2.45, 1.58]
Anxious mood	−2.8 (3.71)	−2.8 (3.26)	[-2.25, 2.79]

We examined changes in the subitems of the Mood Inventory before and after viewing the video, as well as between before and after the online conversation, and similar trends were observed in both groups. The changes in willingness to converse from before viewing the video to before the online conversation were –0.1 ± 1.71 in the intervention group and –0.4 ± 1.40 in the control group (Table 4). The percentage of participants who showed an increased willingness to converse before the online conversation compared to everyday conversation was higher in the intervention group (33.3%, 5/15) than in the control group (13.3%, 2/15).

**Table 4:** Comparison of changes in participants’ willingness to talk before viewing a video and before interacting with the video.

Group	Intervention group	Control group	Difference between the two groups (95% CI)
N	15	15	
mean (sd)	−0.1 (1.71)	−0.4 (1.4)	[-1.22, 0.54]
median (IQR)	0 (−2, 1)	0 (−1, 0)	
(min,max)	(−3, 2)	(−3, 3)	

Participants perceived similarity of the avatar in the viewed video was also higher in the intervention group than in the control group (mean score: 22.5 vs. 14.6, 95% CI = [−13.28, −2.41]), suggesting that participants in the intervention group perceived the avatar as more self-like.

### Feasibility assessment

The study completion rate was high (93.8%, 30/32 participants), demonstrating the feasibility of the intervention. No side effects or adverse events were reported, and all participants who started the intervention completed the scheduled evaluations.

## Discussion

This study is an exploratory randomized controlled study to examine the effectiveness of an intervention using Another Me digital twin technology on participants with social anxiety. For people with social anxiety, first-time conversations can cause strong tension and often lead to avoidance behaviors. Conventional treatments, such as exposure-based cognitive behavioral therapy, are effective [5–7], and a combined approach with video feedback is more favorable because of the reduction in psychological burden. However, these approaches still remain psychologically burdensome [8], and the treatment dropout rate is as high as 15%–25% [30]. In the current study, we examined the effects of video feedback using Another Me before face-to-face conversations with a stranger on reducing tension and anxiety, which were assessed using biophysical monitoring and psychological scales. The results of this study suggest that the pre-dialogue simulation viewing procedure using Another Me digital twin technology may reduce pre-conversation psychophysiological burden and subjective anxiety, rather than demonstrating the isolated effect of Another Me technology itself.

To elucidate the extent to which the participants felt stressed during face-to-face conversations with strangers, we set the primary endpoint as the change in heart rate before and after the online face-to-face conversation. In the main analysis, the between-group difference in heart rate change was evaluated using analysis of covariance (ANCOVA) with sex as acovariate, and 95% confidence interval were calculated. In addition, 95% confidence intervals were calculated for within-group changes before and after the conversation. As secondary analyses, we descriptively examined heart rate at four time points to explore time course pattens.

In the main analysis of the primary endpoint, the heart rate changed by +0.8 ± 7.87 beats/min in the intervention group and by –2.2 ± 5.29 beats/min in the control group before and after the online conversation. The primary endpoint was not met; therefore, no statistically significant between-group difference in heart rate change during the conversation was demonstrated. In the secondary analysis, the heart rate after viewing the video showed a change of –1.7 ± 5.38 beats/min in the intervention group, compared to –0.5 ± 4.76 beats/min in the control group. This effect continued until the online conversation, with a sustainable decrease (–1.5 ± 0.72 beats/min) in the intervention group. In contrast, the control group showed an increase in heart rate (+1.3 ± 6.67 beats/min) between before viewing the video and before the online conversation, with the absolute value of heart rate before the online conversation being higher in the control group.

The secondary endpoints were changes in blood pressure, subjective anxiety and tension (SUDs), mood questionnaires, and willingness to converse. For secondary endpoints, the intervention group showed greater reductions in anxiety (–1.8 ± 3.23 vs. –0.5 ± 1.41) and tension (–1.2 ± 3.23 vs. 0 ± 1.73) after viewing the video than the control group. Furthermore, in the intervention group, the proportion of participants who were more motivated to converse than in their daily lives before the online conversation was higher (33.3% vs. 13.3%). Blood pressure measurements showed that the change in systolic blood pressure between before and after the conversation was minimal in the intervention group (0.0 ± 8.59 mmHg) compared to the control group (+2.3 ± 5.97 mmHg).

### Changes in psychophysiological responses

#### Heart rate change patterns.

The primary endpoint analysis showed that the change in heart rate was relatively small in the intervention group (+0.8 ± 7.87 beats/min) between before and after the online conversation. In contrast, a larger change was seen in the control group (–2.2 ± 5.29 beats/min). We hypothesized that viewing an Another Me video would help relieve tension beforehand and that participants could join the online conversation with a greater sense of calm, which would lead to a stable heart rate during the conversation. Therefore, heart rate changes before and after the online conversation were defined as the main outcome, and we expected that participants in the Another Me group would show slight fluctuation in heart rate when they felt low psychological stress during the conversation with a stranger.

In a study by Rösler et al. [31], the relationship between social anxiety and heart rate was clarified, and it was reported that heart rate increases significantly in a conversation with a stranger. Similarly, Schulz et al. [32] reported that individuals with high social anxiety demonstrated significant heart rate elevation in social situations. In our study, the participants in the control group had greater anxiety and stress before the conversation because of upcoming conversations with strangers, which might have led to an increase in heart rate before the conversation. Finally, they were relieved that the conversation was over, leading to a subsequent reduction in heart rate, and this psychological change might be identified as the fluctuation in heart rate before and after the conversation. A limitation of this study is that we did not continuously measure heart rate during the conversation. Thus, we have no evidence of increased heart rate during the conversation in the control group, as observed in previous studies [31,32].

The secondary analysis results showed that the intervention group had a decreased heart rate after viewing the video, and this effect continued until the completion of the online conversation. In contrast, the control group’s heart rate decreased relatively little after viewing the video and increased before the online conversation. Both groups had similar levels of social anxiety at the beginning of the study. Individuals with high social anxiety have significantly higher heart rates in social situations [32], and increased heart rate during social interactions is believed to be a key feature of social anxiety [31]. As observed in our study, the increased heart rate before conversation in the control group might be a feature of social anxiety, and the reduction in heart rate observed in the intervention group was consistent with well-regulated physiological responses in patients with social anxiety, as reported by McEvoy et al. [33].

#### Changes in subjective anxiety and tension.

The SUDs evaluation showed that anxiety and tension decreased in the intervention group after participants viewed the video, and that they remained lower until the conversation had been completed compared to the control group. Mauss et al. reported a relationship between subjective anxiety and physiological responses in individuals with high social anxiety [34]. Our study supports the link between heart rate changes and subjective anxiety, consistent with the findings of Papageorgiou’s study [35]. Nelson et al. showed a correlation between heart rate, stress levels, and anxiety, which supports the consistency between heart rate and the subjective assessments observed in our study [36].

#### Willingness to converse.

In the intervention group, more participants showed an increased willingness to converse before the conversation compared to the control group (five in the intervention group and two in the control group). Previous research on social anxiety has focused on negative aspects, such as tension and anxiety, and few studies have focused on the positive aspects of willingness to converse. These results provide a new and positive perspective on social anxiety interventions.

#### Effects of Another Me Video.

Several previous studies have supported the effectiveness of video feedback. Harvey et al. [11] reported that video feedback improved negative self-perception in patients with social anxiety. Warnock-Parkes et al. also showed that encouraging appropriate perspective-taking facilitates the correction of distorted self-images [10]. Similar to traditional video feedback, viewing Another Me videos may have allowed participants to objectively observe how they behaved in conversations with others and might have facilitated the correction of distorted self-perceptions. Furthermore, with traditional video feedback, there have been concerns that video feedback does not always work well when participants struggle to behave as they think they should. A key feature of Another Me videos is that they reflect smooth conversations with others and can promote the development of a positive image of successful interactions. Orr et al. reported that video feedback facilitates positive predictions of future performance, which supports the increased willingness to converse observed in this study [13].

An additional issue in interpreting these findings is the validity of Another Me as a person-specific digital representation. Another Me was generated from facial photographs, short speech samples, and profile information. Prior studies showed that its dialogue model generated utterances closer to participants’ own utterances than a general-purpose language model [37], and that its zero-shot speech synthesis achieved higher speaker similarity than conventional methods [36]. Subjective perceived similarity of the avatar was also significantly higher in the intervention group than in the control group. These findings suggest that Another Me achieved some degree of person-specific fidelity. However, a validated Japanese version of the measure used to assess avatar identification was not available, and thus these findings should be interpreted with caution. Furthermore, the present trial did not directly validate the integrated Another Me system against each participant’s actual conversational behavior in matched interactional settings. Therefore, the present findings should be interpreted as supporting the usefulness of a self-relevant and self-identifiable digital pre-exposure stimulus, rather than as proof that a fully equivalent digital twin of the participant had been established.

Even without full validation as an equivalent digital twin, such an approach may still have important clinical value. Although cognitive behavioral therapy-based exposure therapy is effective for social anxiety [5–7], it comes with cost and ethical concerns, personal and structural challenges, and difficulties in controlling stimuli [9].

Additionally, despite the various harmful effects of social anxiety, few people seek professional treatment [2,38]. Inhibitory factors to receiving treatment include financial issues, fear of other individuals’ reactions, and lack of knowledge about where to go for treatment [3]. Interventions for social anxiety using digital twin technology do not require direct contact with others, which are feared by individuals with social anxiety, potentially leading to greater participant safety and a minimal psychological burden. In exposure therapy, participants are required to face the feared stimulus directly, which can lead to increased anxiety and fear. Digital twin technology allows for simulated interactions without contact between participants and the feared subject. This approach offers the possibility to reduce anxiety and fear.

### Limitations

This study had several limitations. First, we did not continuously measure heart rate during conversation. Thus, we found no evidence of increased heart rate during the conversation in the control group, as observed in previous studies [31,32]. Second, although prior studies and subjective ratings supported some degree of person-specific fidelity of Another Me, we did not perform an end-to-end validation of the integrated system against each participant’s actual conversational behavior in matched settings Third, we recruited young adults, mainly women, which limited the generalizability of the results. Fourth, issues have arisen regarding the sustainability of these effects. In our study, only short-term effects were evaluated, and long-term effects have not yet been verified. Fifth, the extent to which the results obtained in this study can be generalized to real-life situations remains unclear. Sixth, the primary measurement method was changed during the study from continuous heart rate monitoring using a smartwatch to pulse rate measurement using an upper-arm blood pressure monitor because of technical issues. Although this change was approved by the ethics committee prior to data analysis, the use of intermittent pulse measurements may have reduced sensitivity for detecting transient physiological changes and may have weakened the evidentiary strength of the primary endpoint. These limitations must be considered when interpreting the results of this study. Seventh, although the sample size was determined using a confidence interval–based precision approach, we didn’t perform formal power calculation because this was first study to examine the efficacy of Another me in human participants. Eighth, since the primary endpoint was to observe the changes in heart rate before and after the interaction, we analyzed the changes only between these two time points using ANCOVA; however, the use of a mixed-effects model might be a more statistically rigorous approach for analyzing repeated measurements over time.

## Conclusion

In this exploratory randomized trial, the pre-dialogue simulation viewing procedure using the “Another Me” digital twin technology did not achieve statistical significance for the primary endpoint, defined as the change in heart rate during the online conversation. However, exploratory secondary analyses suggested potential reductions in pre-conversation heart rate and subjective anxiety and tension, as well as a possible enhancement of motivation to engage in dialogue.

The intervention may have alleviated anticipatory anxiety and tension prior to an initial face-to-face conversation and may have increased willingness to communicate. Although these findings should be interpreted cautiously as hypothesis-generating, they suggest that pre-dialogue simulation using digital twin technology could represent a novel preparatory approach for reducing anticipatory anxiety.

Further large-scale studies incorporating validated assessments of digital twin fidelity and continuous physiological monitoring are required to confirm the clinical significance and underlying mechanisms of this approach.

This method may provide a conceptual foundation for the development of innovative interventions in the treatment of social anxiety symptoms.

## Supporting information

S1 Fig Examples of avatars created. (TIF)

S2 Fig Example of a generated Another Me video. (TIF)

S1 Checklist CONSORT-Checklist. (DOCX)

S1 File Creating a Digital Twin. (DOCX)

S2 File Original protocol (ethics-approved before trial began). (DOCX)

S3 File Amendment 1. (DOCX)

S4 File Amendment 2. (DOCX)

## References

[1] Hofmann SG, Heinrichs N, Moscovitch DA. The nature and expression of social phobia: Toward a new classification. Clin Psychol Rev. 2004;24(7):769–97. doi: 10.1016/j.cpr.2004.07.004 15501556

[2] Crome E, Grove R, Baillie AJ, Sunderland M, Teesson M, Slade T. DSM-IV and DSM-5 social anxiety disorder in the Australian community. Aust N Z J Psychiatry. 2015;49(3):227–35. doi: 10.1177/0004867414546699 25122449 PMC4361462

[3] Olfson M, Guardino M, Struening E, Schneier FR, Hellman F, Klein DF. Barriers to the treatment of social anxiety. Am J Psychiatry. 2000;157(4):521–7. doi: 10.1176/appi.ajp.157.4.521 10739410

[4] Stein MB, Stein DJ. Social anxiety disorder. Lancet. 2008;371(9618):1115–25. doi: 10.1016/S0140-6736(08)60488-2 18374843

[5] Craske MG, Kircanski K, Zelikowsky M, Mystkowski J, Chowdhury N, Baker A. Optimizing inhibitory learning during exposure therapy. Behav Res Ther. 2008;46(1):5–27. doi: 10.1016/j.brat.2007.10.003 18005936

[6] National Institute for Health and Care Excellence. Social anxiety disorder: recognition, assessment and treatment (CG159) clinical guideline. 2013. http://www.nice.org.uk/guidance/cg159 39960994

[7] Mayo-Wilson E, Dias S, Mavranezouli I, Kew K, Clark DM, Ades AE, et al. Psychological and pharmacological interventions for social anxiety disorder in adults: A systematic review and network meta-analysis. Lancet Psychiatry. 2014;1(5):368–76. doi: 10.1016/S2215-0366(14)70329-3 26361000 PMC4287862

[8] Benbow AA, Anderson PL. A meta-analytic examination of attrition in virtual reality exposure therapy for anxiety disorders. J Anxiety Disord. 2019;61:18–26. doi: 10.1016/j.janxdis.2018.06.006 30646997

[9] Neudeck P, Einsle F. Dissemination of exposure therapy in clinical practice: How to handle the barriers? Neudeck P, Wittchen H-U. Exposure therapy: Rethinking the model—Refining the method. 1st ed. New York: Springer Science+Business Media; 2012. 23–34. doi: 10.1007/978-1-4614-3342-2_3

[10] Warnock-Parkes E, Wild J, Stott R, Grey N, Ehlers A, Clark DM. Seeing is believing: Using video feedback in cognitive therapy for social anxiety disorder. Cogn Behav Pract. 2017;24(2):245–55. doi: 10.1016/j.cbpra.2016.03.007 29033532 PMC5627505

[11] Harve AG, Clark DM, Ehlers A, Rapee RM. Social anxiety and self-impression: Cognitive preparation enhances the beneficial effects of video feedback following a stressful social task. Behav Res Ther. 2000;38(12):1183–92. doi: 10.1016/s0005-7967(99)00148-5 11104182

[12] Kim H-Y, Lundh L-G, Harvey A. The enhancement of video feedback by cognitive preparation in the treatment of social anxiety. A single-session experiment. J Behav Ther Exp Psychiatry. 2002;33(1):19–37. doi: 10.1016/s0005-7916(02)00010-1 12389797

[13] Orr EMJ, Moscovitch DA. Learning to re-appraise the self during video feedback for social anxiety: Does depth of processing matter?. Behav Res Ther. 2010;48(8):728–37. doi: 10.1016/j.brat.2010.04.004 20462568

[14] Parr CJ, Cartwright-Hatton S. Social anxiety in adolescents: The effect of video feedback on anxiety and the self-evaluation of performance. Clin Psychol Psychother. 2009;16(1):46–54. doi: 10.1002/cpp.599 19123484

[15] Rodebaugh TL, Heimberg RG, Schultz LT, Blackmore M. The moderated effects of video feedback for social anxiety disorder. J Anxiety Disord. 2010;24(7):663–71. doi: 10.1016/j.janxdis.2010.04.007 20471783 PMC2922463

[16] Kahn JS, Kehle TJ, Jenson WR, Clark E. Comparison of cognitive-behavioral, relaxation, and self-modeling interventions for depression among middle-school students. Sch Psych Rev. 1990;19:196–211. doi: 10.1080/02796015.1990.12085457

[17] Rickards-Schlichting KA, Kehle TJ, Bray MA. A self-modeling intervention for high school students with public speaking anxiety. J Appl Sch Psychol. 2004;20:47–60. doi: 10.1300/J370v20n02_04

[18] Bartholomay EM, Houlihan D. Treating public speaking anxiety: A comparison of exposure and video self-modeling. Int J Psychol Stud. 2018;10:1–10. doi: 10.5539/ijps.v10n4p1

[19] Dowrick PW. A review of self-modeling and related interventions. Appl Prev Psychol. 1999;8:23–39. doi: 10.1016/S0962-1849(99)80009-2

[20] Ishii Y, Ishii R, Lidwina A, Matsuo K, Otsuka A. Support for building relationships in speed dating through observation of pre-dialogue simulations using digital twins. In: Proceedings of the 20th IFIP TC13 International Conference on Human-Computer Interaction (INTERACT), Minas Gerais, Brazil, 2025.

[21] Fresco DM, Coles ME, Heimberg RG, Liebowitz MR, Hami S, Stein MB, et al. The Liebowitz Social Anxiety Scale: A comparison of the psychometric properties of self-report and clinician-administered formats. Psychol Med. 2001;31(6):1025–35. doi: 10.1017/s0033291701004056 11513370

[22] Asakura S, Inoue S, Sasaki F, Asaki Y, Kitagawa N, Inoue T, et al. Reliability and validity of Liebowitz Social Anxiety Scale (LSAS). Japanese version. Clin Psychiatry. 2002;44:1077–84.

[23] Sakurai S, Sakurai T. Construction of Japanese version of shyness scale for college students. Bull Nara Univ Educ. 1991;40:235–43.

[24] Sakano Y, Fukui T, Kumano H, Horie H, Kawahara K, Yamamoto H. Development and validation of a new mood inventory. Jpn J Psychosom Med. 1994;34:629–36.

[25] Wolpe J. Psychotherapy by reciprocal inhibition. Stanford, CA: Stanford University Press. 1958.

[26] Van Looy J, Courtois C, De Vocht M, De Marez L. Player identification in online games: Validation of a Scale for Measuring Identification in MMOGs. Media Psychology. 2012;15(2):197–221. doi: 10.1080/15213269.2012.674917

[27] Kito K, Suzuki K. Effect of foot massage and foot bath for healthy college students influence to autonomic nerve activity and mood. Bull Meio Univ Res Inst. 2016;25:151–6.

[28] Hopewell S, Chan A-W, Collins GS, Hróbjartsson A, Moher D, Schulz KF, et al. CONSORT 2025 statement: Updated guideline for reporting randomised trials. BMJ. 2025;389:e081123. doi: 10.1136/bmj-2024-081123 40228833 PMC11995449

[29] Hopewell S, Chan AW, Collins GS, Hróbjartsson A, Moher D, Schulz KF. The CONSORT reporting checklist. Harwood J, Albury C, Beyer J, Schlüssel M, Collins G. The EQUATOR network reporting guideline platform. The UK EQUATOR Centre. 2025.

[30] Kampmann IL, Emmelkamp PMG, Hartanto D, Brinkman W-P, Zijlstra BJH, Morina N. Exposure to virtual social interactions in the treatment of social anxiety disorder: A randomized controlled trial. Behav Res Ther. 2016;77:147–56. doi: 10.1016/j.brat.2015.12.016 26752328

[31] Rösler L, Göhring S, Strunz M, Gamer M. Social anxiety is associated with heart rate but not gaze behavior in a real social interaction. J Behav Ther Exp Psychiatry. 2021;70:101600. doi: 10.1016/j.jbtep.2020.101600 32882674 PMC7689581

[32] Schulz SM, Alpers GW, Hofmann SG. Negative self-focused cognitions mediate the effect of trait social anxiety on state anxiety. Behav Res Ther. 2008;46(4):438–49. doi: 10.1016/j.brat.2008.01.008 18321469 PMC2430992

[33] McEvoy PM, Hyett MP, Johnson AR, Erceg-Hurn DM, Clarke PJF, Kyron MJ, et al. Impacts of imagery-enhanced versus verbally-based cognitive behavioral group therapy on psychophysiological parameters in social anxiety disorder: Results from a randomized-controlled trial. Behav Res Ther. 2022;155:104131. doi: 10.1016/j.brat.2022.104131 35696837

[34] Mauss IB, Wilhelm FH, Gross JJ. Is there less to social anxiety than meets the eye? Emotion experience, expression, and bodily responding. Cogn Emot. 2004;18:631–42. doi: 10.1080/02699930341000112

[35] Papageorgiou C, Wells A. Effects of heart rate information on anxiety, perspective taking, and performance in high and low social-evaluative anxiety. Behavior Therapy. 2002;33(2):181–99. doi: 10.1016/s0005-7894(02)80024-7

[36] Nelson BW, Harvie HMK, Jain B, Knight EL, Roos LE, Giuliano RJ. Smartphone photoplethysmography pulse rate covaries with stress and anxiety during a digital acute social stressor. Psychosom Med. 2023;85(7):577–84. doi: 10.1097/PSY.0000000000001178 37409791

[37] Maekawa K. Corpus of spontaneous Japanese: Its design and evaluation. SSPR. 2003.

[38] Magee WJ, Eaton WW, Wittchen HU, McGonagle KA, Kessler RC. Agoraphobia, simple phobia, and social phobia in the National Comorbidity Survey. Arch Gen Psychiatry. 1996;53(2):159–68. doi: 10.1001/archpsyc.1996.01830020077009 8629891

