# Supplementary material for: Investigating “Another Me” digital twin technology to support the development of human relationships: An exploratory randomized controlled study
Source: PLoS One. 2026 Aug 7;21(8):e0353835. doi: 10.1371/journal.pone.0353835 (PMC13450786; doi:10.1371/journal.pone.0353835)
Supplement: Supplementary file 2 [file pone.0353835.s004.docx]

**S1 Text. Creating a Digital Twin**

Facial photographs, several seconds of speech sounds, profile information (nickname, age, sex, location, occupation, hobbies), avatar hairstyle, and avatar equipment were collected from the participants in the intervention group (Supplementary Table 1).

**Supplementary Table 1. Examples of profile and avatar information**

Cartoon-style avatars were created from facial photographs using a customized Motion Portrait SDK from AX Inc. The SDK functioned solely as a software tool to process the images, without using pre-existing graphic materials. Thus, the resulting avatars are fully original. (S1 Fig). The participants specified hairstyles and equipment.

**S1 Fig. Examples of avatars created.** Examples of avatars generated from images

The individual shown in the image is a co-author of this manuscript and provided written informed consent for publication using the PLOS consent form. The photograph was created originally by authors to provide representative image which was not obtained from specific study participant.

We also created a speech synthesis model to generate arbitrary speech from speech utterances while reproducing individual speech features using zero-shot text-to-speech synthesis technology [39]. This can be achieved by separating the rhythm- and acoustic-feature-based speaker characteristics and obtaining embedding vectors from the pre-trained model through self-supervised learning. Self-supervised learning can reproduce individual characteristics more accurately by obtaining the embedding vectors from a pre-trained model. In the experiment, a speech synthesis model learned from the speech data of 1,083 participants and an SSL model [37] learned from the speech data of 1,417 participants was used to generate a speech synthesis model from a short 10–15 s speech for each participant. Furthermore, we created a speech-generation model (Persona Pair Prompt Dialogue Model) that can generate speech based on profile information from both profiles [40,41]. This is a pre-trained (transformer encoder–decoder model, with 222M parameters) using a dialogue log consisting of Twitter reply pairs and profile information estimated from reply contents [42]. The model is a pre-trained transformer encoder–decoder model with 222M parameters. In the experiment, the profile information of the participant and experimenter, including the standardized utterances from the participant to the experimenter (see below), were used as prompts to generate speech content. These were combined to create a digital twin that reproduced an individual’s appearance, voice, and speech. For the control group, the digital twin was created using data from the experimenter rather than from the participant.

**Creating Another Me Video**

In the intervention group, a dialogue video (Another Me videos) was created between the participant’s own digital twin and the digital twin of the online conversation partner described below. In the control group, dialogue videos were made between two experimenters (neither was an online conversation partner). The dialogue content was generated by adjusting the prompts to the persona dialogue model so that the flow would be as follows (1. through 3.).

1. Give the person’s name and greet them.
2. Immediately after the greeting, and alternately every seven or eight remarks thereafter, please select a topic from the hobbies in the other person’s profile information that is similar in meaning to your hobbies, in that order, and mention them.
3. Once you have discussed their hobbies, ask if they have anything else to discuss.

For the selection of hobbies with similar meanings in 2, “topics that are exciting” were selected and ranked by ChatGPT GPT-4 from the information in the hobby lists of both parties.

For the generated speech content, if a dialogue broke down owing to meaningless utterances or repetition of the same utterance, or if an utterance that was not true was repeated, the dialogue was discarded, and the speech content was regenerated. Examples of the generated dialogues are presented below.

Person A: Hello E.

Person B: Hello, Mr. T. Nice to meet you!

Person A: I see that gaming is your hobby. I am interested in it, too.

Person B: Yes, I love games. I especially like quizzes, and I play them a lot!

Person A: I see! I love games, especially RPG and action games.

Person B: RPG or action game? That sounds interesting. What kinds of games do you like?

Person A: I love RPGs, especially the Final Fantasy series. I think the stories are deep, and the characters are fascinating!

Person B: I know the Final Fantasy series, too. The story is certainly fascinating!

Person A: Yes, it is! I think Final Fantasy Vii is a masterpiece among masterpieces. The story is so fascinating that I keep playing it repeatedly, lol!

Person B: On a different note, skiing is also your hobby. It sounds very intriguing.

Person A: Yes, I love skiing and go about twice per week. There are many ski resorts in Kanagawa Prefecture!

The avatar’s movements during the speech were generated using an automatic body-motion generation method [43]. It uses a pre-trained model learned from 28 dialogues of 280 min between 11 pairs of Japanese people in their 20s–50s who had never met each other before. The model generates eye movements, head direction, nodding, hand gestures, and upper-body posture using the generated speech content as input. The Big Five characteristic scores were not input for generating body motions.

Using the speech content, speech-synthesis models, avatar data, and body-motion data generated from the above, Epic Games Inc. reproduced the dialogue in the Unreal Engine 5 virtual space and recorded it as a dialogue video. The generated Another Me video lasted for an average of 4 min 30 s (standard deviation of 23 s) (S2 Fig).

**S2 Fig. Example of a generated Another Me video.** Example video of Another Me

The individuals shown in the image are co-authors of this manuscript and provided written informed consent for publication using the PLOS consent form. The photograph was created originally by authors to provide representative image which was not obtained from specific study participants.

# **References**

39. Fujita K, Ashihara T, Kanagawa H, Moriya T, Ijima Y. Zero-shot text-to-speech synthesis conditioned using self-supervised speech representation model. In Proceedings of the 2023 IEEE International Conference on Acoustics, Speech, and Signal Processing Workshops (ICASSPW); 2023, Jun 4-10; Rhodes Island, Greece. IEEE; 2023. pp. 1-5.
40. Otsuka A, Matsuo K, Ishii R, Nomoto N, Sugiyama H. User-Specific Dialogue Generation with User Profile-Aware Pre-Training Model and Parameter-Efficient Fine-Tuning. arXiv:2409.00887 [Preprint]. 2024 September 2 [cited 2025 February 13]. Available from: <https://arxiv.org/abs/2409.00887>
41. Otsuka A, Ishii R, Nomoto N, Sugiyama H, Fukayama A, Nakamura T. A Study on Pre-Training Based Open Domain Dialogue Model with User Profiles toward Reproducing Individuality. In The 96^th^ SIG-SLUD; 2022, Dec 13-14; Tokyo, Japan. The Japanese Society for Artificial Intelligence; 2022. pp. 26.
42. Sugiyama H, Mizukami M, Arimoto T, Narimatsu H, Chiba Y, Nakajima H, et al. Empirical analysis of training strategies of transformer-based Japanese chit-chat systems. In Proceedings of the 2022 IEEE Spoken Language Technology Workshop (SLT); 2023, Jan 9-12; Doha, Qatar, IEEE; 2023. pp. 685-691.
43. Ishii R, Ahuja C, Nakano Y, Morency LP. Impact of Personality on Nonverbal Behavior Generation. In Proceedings of the 20th ACM International Conference on Intelligent Virtual Agents (IVA '20). Association for Computing Machinery, New York, NY, USA, Article, 2020. 29, 1-8.
