# Supplementary material for: Investigating “Another Me” digital twin technology to support the development of human relationships: An exploratory randomized controlled study
Source: PLoS One. 2026 Aug 7;21(8):e0353835. doi: 10.1371/journal.pone.0353835 (PMC13450786; doi:10.1371/journal.pone.0353835)
Supplement: Supplementary file 4 [file pone.0353835.s006.docx]

研究計画書

研究課題名:

Another Meによる人間関係構築支援の検証について

研究責任者：

横浜市立大学　研究・産学連携推進センター　宮﨑 智之

版数：第2.0版

作成年月日：2024年3月11日

改訂履歴

| 版数 | 作成/改訂日 |
| --- | --- |
| 第1版 | 2023年12月26日 |
| 第2版 | 2024年3月11日 |
|  |  |
|  |  |

**本学の臨床研究に関する個人情報の利用目的について**

　本学は、個人情報の保護に関する法律（平成15年法律第57号、以下「個情法」という。）第16条第8項に規定される「学術研究機関等」に該当し、大学その他の学術研究を目的とする機関である。

本学で実施する臨床研究は、個情法第18条第3項第5号及び第6号に規定される個人情報及び個人データについて学術研究目的で取り扱われる。

目次

[1.	本研究の目的	6](#_Toc80372039)

[2.	研究の背景と意義	6](#_Toc80372040)

[2.1.	研究の背景	6](#_Toc80372041)

[2.2.	研究の意義	7](#_Toc80372042)

[2.3.	科学的合理性の根拠	7](#_Toc80372043)

[3.	研究対象者	8](#_Toc80372044)

[3.1.	選択基準	8](#_Toc80372045)

[3.2.	除外基準	8](#_Toc80372046)

[4.	研究計画	9](#_Toc80372047)

[4.1.	研究デザイン	9](#_Toc80372048)

[4.2.	予定する研究対象者数	9](#_Toc80372049)

[4.3.	研究期間	9](#_Toc80372050)

[5.	研究の方法	9](#_Toc80372051)

[5.1.	観察・検査項目	9](#_Toc80372052)

[5.2.	評価項目	13](#_Toc80372053)

[5.3.	統計解析方法	14](#_Toc80372054)

[6.	インフォームド・コンセントを受ける手続	15](#_Toc80372055)

[7.	研究対象者の登録方法	15](#_Toc80372056)

[8.	研究参加の中止基準	16](#_Toc80372057)

[9.	試料・情報の取扱い	16](#_Toc80372058)

[9.1.	個人情報の加工の方法	16](#_Toc80372059)

[9.2.	試料／情報の授受	16](#_Toc80372060)

[9.3.	試料・情報の保管方法及び保管期間	17](#_Toc80372061)

[9.4.	試料・情報の廃棄方法	18](#_Toc80372062)

[9.5.	試料・情報の提供の記録	18](#_Toc80372063)

[9.6.	試料・情報の二次利用	18](#_Toc80372064)

[10.	個人情報の保護	19](#_Toc80372065)

[10.1.	個人情報の取扱い	19](#_Toc80372066)

[10.2.	個人情報の開示等への対応	19](#_Toc80372067)

[10.3.	個人情報の取扱い等に関する問合せ及び苦情への対応	19](#_Toc80372068)

[11.	研究結果（偶発的所見を含む）の取り扱い	19](#_Toc80372069)

[11.1.	研究結果の開示方針	19](#_Toc80372070)

[11.2.	偶発的所見の取り扱い方針	20](#_Toc80372071)

[11.3.	研究対象者への説明方法	20](#_Toc80372072)

[11.4.	遺伝カウンセリングの体制	20](#_Toc80372073)

[12.	研究対象者に生じる負担並びに予測されるリスク及び利益	20](#_Toc80372074)

[12.1.	利益	20](#_Toc80372075)

[12.2.	負担・リスク及びそれらを最小化する対策	20](#_Toc80372076)

[13.	健康被害に対する補償	20](#_Toc80372077)

[14.	研究対象者の費用負担・謝礼	21](#_Toc80372078)

[15.	研究の資金源	21](#_Toc80372079)

[16.	利益相反	21](#_Toc80372080)

[17.	研究実施にかかる手続	21](#_Toc80372081)

[17.1.	遵守すべき諸規則	21](#_Toc80372082)

[17.2.	倫理審査委員会への付議	21](#_Toc80372083)

[17.3.	研究機関の長による許可等	22](#_Toc80372084)

[17.4.	定期報告	22](#_Toc80372085)

[17.5.	研究の停止・中止	22](#_Toc80372086)

[17.6.	研究の終了	22](#_Toc80372087)

[18.	モニタリング及び監査	22](#_Toc80372088)

[19.	研究の情報公開及び結果の公表	22](#_Toc80372089)

[20.	研究成果の帰属	22](#_Toc80372090)

[21.	研究対象者及びその関係者からの相談等への対応	23](#_Toc80372091)

[22.	研究に関する業務の委託	23](#_Toc80372092)

[23.	研究実施体制	23](#_Toc80372093)

[23.1.	研究責任者	23](#_Toc80372094)

[23.2.	既存試料・情報の提供のみを行う機関と責任者	23](#_Toc80372096)

[23.3.	研究事務局	23](#_Toc80372097)

[23.4.	統計解析責任者	24](#_Toc80372098)

[23.5.	データセンター及びマネジメント責任者	24](#_Toc80372099)

[24.	引用文献	24](#_Toc80372100)

**略語及び用語の定義**

本研究計画書に使用する略語及び用語を下記に示す。

| 略語 | 正式名称 | 説明 |
| --- | --- | --- |
| FAS | Full analysis set | 最大解析対象集団 |
| iOSDMH | Implementation Outcome Scales for Digital Mental Health | プログラム評価尺度 |
| LSAS | Liebowitz Social Anxiety Scale, Self-Report | 社交不安症の評価スケール |
| PPS | Per Protocol Set of Subject | 研究計画書適合例 |
| SNS | Social Networking Service | ソーシャルネットワーキングサービス |
| SST | Social Skills Training | 社会的スキル訓練 |
|  |  |  |

# 1. 本研究の目的

社交不安や対人不安を抱える方の問題として、うまく対話できるイメージを作り出せない、自己に対するネガティブなイメージに固執し他者の目を過度に気にする傾向がある。本研究では、対話再現を用いることで対人不安の低減が可能かという仮説の下、自身のAnother Me（自分自身を模した自律的に動くアバター・キャラクタ）と他者とのコミュニケーションを視聴することで、オンライン対話時の緊張や不安が緩和される可能性について調べることを目的としている。

# 2. 研究の背景と意義

## 2.1. 研究の背景

シャイネスは対人不安の一形態で、Jones and Russellによる定義^1)^によれば「他者との適切な交流を阻害する種類の対人不安」とされる。初対面の状況やスピーチ、会話など、他者との対話の際に生じる緊張や不安は、対人不安を感じる人々が他者とのコミュニケーションに苦しむ原因となり、社会的不適応に陥るリスクを高める^2)^。このような人々が学校や職場などの様々な社会的場面で困難に直面しているという報告が存在する^3)^。シャイネスは精神的苦痛だけでなく、日常生活の障壁となり、生活の質（QOL）を低下させると指摘されている^4)^。

現代社会ではニートや引きこもりなどの社会適応に困難を抱える若者の数が増えており、厚生労働省（2007）はそのような若者の約80％が対人関係に不安を感じ、対人恐怖が、その一因であることを指摘している^5)^。安井らは軽度の対人恐怖の増加に着目し、これが社会的不適応に繋がる可能性を述べている^6)^。Learyはシャイネスを「対人的評価に直面する、あるいはその予期から生じる対人不安や行動の抑制によって特徴づけられる情動的－行動的症候群」と定義し、社会的場面での不適応との関連を述べている^7)^。

特に近年、COVID-19の影響で普及したテレワーク、オンライン授業、そして学会のオンライン開催が、対人不安を抱えていた人々にとって社会参加の機会を拡げたとされている^2)^。シャイネスが高い人々は、従来の研究によっても、対面でのコミュニケーションよりもメールやSNSを利用したComputer Mediated Communicationの方が、対話がしやすいと感じる傾向があることが示されていた^8,9)^。さらに、対人不安が高い大学生の中には、対面での講義よりオンラインでの講義を好むという声が報告されている^10)^。この急激なデジタル化へのシフトは、そうした者たちにとってプラスの影響を与え、社会参加と社会復帰へつながることが期待される。

また、シャイネスは社会的な不適応に繋がり、社会的スキル訓練(SST)が必要とされているが、その効果は一様ではない。自己認知やメタ認知の修正がシャイネスの高い人々のSSTの効果を高める可能性がある。Greshamによれば、スキルの欠如はスキルの獲得・遂行の有無によって分類でき^11)^、シャイネスにおいては獲得しているが遂行できない「実行欠如」にあたる^12)^。

近年、ストレス状態を客観的に評価する方法として、ウェアラブルデバイスを用いて測定可能な心拍変動が注目されている。Computer Mediated Communication体験時にウェアラブルデバイスを用いることにより、主観的な指標だけでなく、客観的な指標も参考に、VR体験中に対象者が感じるストレスの状態をより詳細に評価することが可能となる。

## 2.2. 研究の意義

1. 社会的意義:

- 社会参加の促進: 対人不安を抱える者が、リアルな社会参加の前段階として仮想空間における社会的インタラクションを経験することで、対人関係の不安を減少させることが可能となる。これは、社会復帰や社会活動への円滑な移行を促すものと考えられる。
- 自己理解の促進: 個人のコミュニケーションパターンの可視化により、ユーザは自己認識を深め、自己改善の機会を得ることが可能である。これにより、自身の弱点や改善点を客観的に理解し、対人関係を強化することが期待される。

1. テクノロジー上の意義:

- パーソナライズされた学習: AIが個々のコミュニケーションスタイルに合わせてカスタマイズされたフィードバックを自動的に提供する。
- 自己認知の向上: コミュニケーションのパターンを可視化し、自身の強みと弱みを理解するのに役立つ。
- 行動変容の促進: 仮想的なやり取りを繰り返すことで、リアルな状況での行動変容が促進される可能性がある。

1. 展望

- アクセシビリティの向上: テクノロジーの普及により、より多くの人がこのツールにアクセスできるようになる。
- データの蓄積と分析: 大量のコミュニケーションデータから得られる洞察を基に、より効果的な治療法や支援プログラムを開発できる。
- エシカルな利用: 個人のプライバシーを守りながら、効果的なコミュニケーション支援を行う方法の開発につながることが期待できる。

## 2.3. 科学的合理性の根拠

　本研究における対象者は、初対面の相手とのコミュニケーション時に過度な緊張を自覚する方である。初対面の状況やスピーチ、会話など他者との対話の際に生じる緊張や不安は、対人不安を感じる人々が他者とのコミュニケーションに苦しむ原因となり、社会的不適応に陥るリスクを高める^2)^。社交不安や対人不安を抱える方の問題として、うまく対話できるイメージを作り出せない、自己に対するネガティブなイメージに固執し他者の目を過度に気にする傾向がある。社交不安や対人不安に対する介入として、社会的スキル訓練(SST)が挙げられるが、その効果は一様ではなく、解決策の探求が求められている。

本研究では自身のAnother Me（自分自身を模した自律的に動くアバター・キャラクタ）の対話再現を視聴することにより、自己のポジティブな側面に注目できるようになると同時に、不一致やギャップがあればそれに気付き、修正の機会を得ることができる。これにより、自身の弱点や改善点を客観的に理解し、対人関係を強化することが期待される。

本研究はランダム化比較試験を採用しており、対象者を介入群と対照群にランダムに割り付けることにより両者の性質の違いが結果に及ぼす可能性が少なく、高い信頼性を持って介入の効果を比較することができる。

# 3. 研究対象者

以下の選択基準をすべて満たし、かつ除外基準のいずれにも該当しない方を対象とする。

## 3.1. 選択基準

以下の基準をすべて満たす方を対象とする。

1) 初対面の人と話すときに緊張してしまうと自覚がある方（あがり症）^※^
2) 18歳以上の一般成人
3) みなとみらいオフィスへの訪問が可能な方
4) 本研究の参加に関して同意が文書で得られる方
5) 日常的にガーミンウォッチを装着することが可能な方

※：社交不安症の評価スケール、シャイネス尺度日本語版等による診断結果による判定ではなく、研究対象者本人があがり症であると自覚している方を対象とする。

設定根拠：

1,2)対人不安（シャイネス傾向）を抱える方を対象としているため

3) みなとみらいオフィスにて研究の説明及び同意の取得を行うため
4) 研究対象者への倫理的配慮
5) ガーミンウォッチを装着し心拍数等を計測するため

## 3.2. 除外基準

以下のいずれかに抵触する患者は本研究に組入れない。

1) 日本語による会話が困難な方
2) 心房細動あるいは不整脈の既往を指摘された方

設定根拠：

1) 研究の過程でオンライン対話をおこなうため
2) 心拍数が主要評価項目であるため

# 4. 研究計画

## 4.1. 研究デザイン

本研究は探索的、並行群間、非盲検、ランダム化比較試験である。

## 4.2. 予定する研究対象者数

予定症例数：30例

（内訳：介入群15例、対照群15例）

【設定根拠】

先行研究として、大学生を対象としたリラクセーションの介入では、マッサージ前後で心拍数が平均2.6回/分減少していた。動画視聴による心拍数の減少も同程度と考え、標準偏差を5回/分としたとき、95%信頼区間の信頼区間幅を5.2回/分で得るために必要なサンプルサイズは15であるため、それぞれ15例の計30例とした。

## 4.3. 研究期間

研究期間：研究機関の長の実施許可日～2025年3月31日

予定登録期間：研究機関の長の実施許可日～2024年3月6日

# 5. 研究の方法

## 5.1. 観察・検査項目

［観察・検査項目］

1. 試料

試料の採取は行わない。

1. 情報

　　(1)研究のために新たに収集する情報

背景情報：年齢、性別、所属

精神評価尺度：社交不安症の評価スケール（LSAS）、シャイネス尺度日本語版、気分調査票

生体指標：心拍数、心拍変動、脈拍、血圧

Another Me作成に必要なプロフィール情報：ニックネーム、所在地、趣味、相手との関係、顔写真、音声データ

探索的評価項目：会話への意欲に関するアンケート、アバターの同一性に関するアンケート、不安や緊張に関するアンケート

　　(2)既存情報

　既存情報の取得は行わない

［研究について］

本研究は、ビデオを利用したイメージ形成を支援する手法において、認知行動療法のアプローチを参考にしている。具体的には、以下の二つのビデオベース手法を参考にしている。

1. ビデオ・セルフモデリング：

この手法では、個人の成功行動をビデオ撮影し、その映像を視聴することにより、成功体験のイメージを形成することを目指す。ビデオモデリングを通じて、個体が自己能力に対して肯定的な視点を持つことを促す。

1. ビデオフィードバック：

この方法では、自己の行動や反応をビデオに記録し、その映像を観察することで、自己振る舞いを客観的に評価し、自己認識を促進することを目的とする。これにより、ネガティブな自己イメージと実際の行動との間のギャップに気付き、修正を促す。

更に、研究は「Another Me対話」というコンセプトに関連づけている。このコンセプトは、個人の分身が対話を効果的に行っている様子をビデオで視聴することで、モデリング効果を体験できることを指す。これにより、個人は自己のポジティブな側面に注目できるようになると同時に、不一致やギャップがあればそれに気付き、修正の機会を得ることが可能である。生成された対話の特定の部分を強調または抑制することで、自己認識の向上を図る。

・研究が有効な根拠：NTT社のパイロット研究。研究の中では、事前に相手の情報を取得することで、実際のコミュニケーション時における緊張度が下がり、対話のイメージの具体化がしやすくなったと報告された。また、参加者へのヒアリングでも初対面では共通話題を探す対話戦略を取る人が多かった。

［Another Meについて］

Another Me とは、自らと共存し共に成長するデジタルツインのことである。性格や価値観、対話時の特徴を学習させ、経験を共有することにより共に成長する。

［Another Me対話動画について］

個人の分身が対話を効果的に行っている様子をビデオで視聴することで、モデリング効果を体験できる。これにより、個人は自己のポジティブな側面に注目できるようになると同時に、不一致やギャップがあればそれに気付き、修正の機会を得ることが可能。生成された対話の特定の部分を強調または抑制することで、自己認識の向上を図る。

研究対象者は、5分～10分程度のAnother Me同士の対話動画を視聴する。動画の視聴は研究対象者の自宅で1回のみ行い、1日で終了します。

［割付］

同意取得後、研究対象者を2群（介入群、対照群）にランダムに割り付ける。性別を割付因子とする。

【介入群（Ｎ＝15）】介入群に割り付けられた研究対象者は、NTTデジタルツインコンピューティング研究センタによってAnother Meの作成、Another Me 対話動画の作成が行われる。研究対象者の自宅にて、自身のAnother Me対話動画を視聴し、その後オンライン対話を行う。

【対照群（Ｎ＝15）】対照群に割り付けられた研究対象者は、Another Meの作成は行わない。研究対象者の自宅にて、他者のAnother Me対話動画を視聴し、その後オンライン対話を行う。

［研究手順］

Step1：動画視聴前調査

研究対象者は、webアンケートにて、LSAS、シャイネス尺度日本語版、気分調査票、不安や緊張、会話への意欲に関する質問に回答する。ガーミンウォッチを用いて心拍数と脈拍を、血圧計を用いて血圧を測定する。ガーミンウォッチに関しては、Step6まで継続して測定する。

Step2：Another Me対話動画視聴

介入群は自分と他者BのAnother Me動画を視聴する。対照群は、他者Cと他者DのAnother Me動画を視聴する。

Step3： 動画視聴後調査

webアンケートにて、不安や緊張に関する質問に回答する。血圧を測定する。

Step4：オンライン対話前調査

webアンケートにて、不安や緊張に関する質問、気分調査票、会話への意欲に関する質問に回答する。血圧を測定する。

Step5：オンライン対話

介入群、対照群共に他者Bとオンライン対話を行う。

Step6：オンライン対話終了後調査

webアンケートにて、不安や緊張に関する質問、気分調査票、iOSDMH、アバターの同一性に関する質問に回答する。血圧を測定する。ガーミンウォッチの測定を終了する。

［観察・検査スケジュール］

|  | 評価項目 | 実施  場所 |  | 動画  視聴前 | 動画  視聴中 | 動画  視聴後 | 対話前 | 対話中 | 対話後 |
| --- | --- | --- | --- | --- | --- | --- | --- | --- | --- |
| Day0 | 同意取得 | みなとみらい | 〇 |  |  |  |  |  |  |
|  | 対象者背景の確認 | みなとみらい | 〇 |  |  |  |  |  |  |
|  | プロフィール作成 | みなとみらい | 〇 |  |  |  |  |  |  |
| Day14  いずれか1日 | 心理アンケート | オンライン |  | 〇 |  | 〇 | 〇 |  | 〇 |
|  | 生体モニタリング | 自宅 |  |  |  |  |  |  |  |
|  | 動画視聴 | 自宅 |  |  | 〇 |  |  |  |  |
|  | オンライン対話 | オンライン |  |  |  |  |  | 〇 |  |

## 5.2. 評価項目

### 5.2.1. 主要評価項目

ガーミンスマートウォッチから計測した心拍数。

心拍数をオンライン対話時に経時的に測定し、その変化量を対照群と比較することで、Another Me動画視聴により社交不安や対人不安による緊張や不安への効果を探索的に検討する。

### 5.2.2. 副次的評価項目

・社交不安症の評価スケール(Liebowitz Social Anxiety Scale, Self-Report: LSAS)：社交不安障害の重症度を評価するための尺度である。この尺度は、社交場面での不安や恐怖を数値化し、社交不安障害の診断や治療効果の測定に用いられる。構成は、さまざまな社交的状況における恐怖や回避行動を自己評価する形式で、24項目からなる。各項目では、不安の度合いと回避行動の頻度を評価する。これにより、社交不安の程度を定量的に把握し、治療の進捗を評価することができる。日本語版は、日本の文化や社会的背景を考慮した内容となっている。

・シャイネス尺度（Jones and Russell 1982）：シャイネス尺度（Jones and Russell 1982）日本語版は、内気さや恥ずかしさを測る心理学的尺度の日本語化版。この尺度は自己報告式アンケートで、社交的状況や対人関係での反応や感情を問う項目から構成される。回答者は各項目について自分がどの程度当てはまるかをスケールで答える。日本語版は原版を日本の文化や言語に合わせて翻訳・調整。日本の研究者や臨床家が日本人の内気さや社交的不安を評価し、研究や治療に活用できるようになっている。心理学研究、臨床評価、自己理解のために広く使われている。

・気分調査票：個人の気分や感情状態を評価する心理学的ツールである。様々な感情や感覚を表す質問や文で構成され、回答者が自分の現在の気分や最近の感情状態を評価する形式を取られている。これにより、個人の心理状態や感情の変動を把握でき、心理学研究、臨床診断、治療の進捗評価などに利用されている。

・iOSDMH：Implementation Outcome Scales for Digital Mental Health (iOSDMH)は、デジタルメンタルヘルスプログラムの実装性評価尺度である。オンライン対話後にWebアンケートにより実施する。

・ガーミンウォッチ：心拍数、血中酸素、体温、カロリー等を測定することができる。

・血圧計：心臓が収縮する時の血圧（最高血圧）、心臓が拡張する時の血圧（最低血圧）の２つの数値を測定することができる。動画視聴前後、オンライン対話前後の血圧を測定する。

なおガーミンウォッチ、血圧計は貸与する。貸与時に返送用のキットを配布し、研究対象者は、研究終了後郵送で返却する。

・探索的評価項目

不安や緊張に関するアンケート

会話への意欲に関するアンケート

アバターの同一性に関するアンケート

## 5.3. 統計解析方法

解析対象集団

解析対象集団は，以下のように定義する．主解析はFAS, 副次解析(補足的解析)はPPSとする．

最大解析対象集団(full analysisss set: FAS)

本研究に登録され，ランダム化後にAnother meを視聴した主要評価項目に関するデータがあるすべての研究対象者をFASとする．また，割付と異なる場合でも割付通りに集計解析する．ただし，ベースラインデータが取得できない研究対象者および重大な研究計画書違反(同意未取得，契約期間外登録等)の研究対象者については除外する．

研究計画書に適合した対象集団(per protocol set:PPS)

FASから，研究方法など計画書の規定に対して，以下の重大な違反があった症例を除いた集団をPPSとする．

・選択基準違反

・除外基準違反

### 5.3.1. 主要評価項目の解析

主要な解析

各群のオンライン対話前後の心拍数の変化量を要約する．主たる解析として，各群の心拍数の変化量の95%信頼区間を算出する．

副次的な解析

FASを対象に両群のオンライン対話前後の心拍数の変化量の差の95%信頼区間を算出する．さらに両群の変化量の差の比較をするために，共分散分析を行う．両側検定でp<0.05で統計学的に有意差があると判定する．

### 5.3.2. 副次評価項目の解析

副次評価項目で収集したデータについてはオンライン対話前後の変化に関する群間比較を行う。

# 6. インフォームド・コンセントを受ける手続

本研究は、研究対象者から本研究のために新たに試料・情報を取得して実施する研究である。人を対象とする生命科学・医学系研究に関する倫理指針第8の1（1）に該当するため、研究責任者又は研究分担者は、研究対象者が研究に参加する前に、倫理審査委員会で承認された説明・同意文書を用いて以下の事項を十分に説明する。研究対象者が内容を十分理解したことを確認した後、参加の同意を文書により取得する。

研究対象者が本研究への参加を辞退する場合は、その理由にかかわらず、「同意撤回書」を用いて、研究への参加を中止することができる。その場合は直ちに当該研究対象者の試料・情報は破棄処分する。ただし、同意撤回の申し出の時点ですでに研究結果が公表されていたときなど、データから除けない場合がある。

１． 研究の実施について許可を受けていること

２． 研究機関の名称及び研究責任者の氏名

３． 研究の目的及び意義

４． 研究の方法及び期間

５． 研究の対象者として選定された理由

６． 研究対象者に生じる負担並びに予測されるリスク及び利益

７． 健康被害に対する補償の有無及びその内容

８． 同意を撤回できること

９． 不利益を受けないこと

10．個人情報等の取り扱い

11．試料・情報の保管及び廃棄の方法

12．試料・情報の二次利用について

13．研究により得られた結果等の取り扱い

14．費用及び謝礼について

15．研究の資金源と利益相反

16．研究に関する情報公開の方法

17．資料を閲覧できること

18．関係者が試料・情報を閲覧すること

19．連絡お問い合わせ先

# 7. 研究対象者の登録方法

(1) 研究対象者が適格基準をすべて満たし、本人より研究参加の同意が得られたら、研究責任者又は研究分担者は症例登録票に記載し、症例登録票に必要事項を記載し登録センターにメールで送信する。
(2) 登録センターは、症例登録票の記載内容から適格性を判定し、適格と判定した場合は割付を実施する。なお、割付因子は性別とする。
(3) 登録センターは、登録確認票に割付け結果を記載し、研究責任者又は研究分担者にメールで通知する。
(4) 研究責任者又は研究分担者は登録確認票を保存する。

登録センター

施設名：株式会社ヌーベルプラス データセンター

TEL：03-6680-2525

FAX：03-6866-9618

Mail： jst@n-place.co.jp

受付時間：平日 9 時から 17 時（土日、祝祭日、年末年始は受付けない）

# 8. 研究参加の中止基準

本研究参加の中止基準は、以下のとおりとする。

1） 研究対象者より同意の撤回/研究利用への拒否の連絡があった場合
2） 研究責任者が研究の継続が困難と判断した場合
3） 登録後に選択・除外基準違反が判明した場合

# 9. 試料・情報の取扱い

## 9.1. 個人情報の加工の方法

研究責任者又は研究分担者は、研究対象者に対して研究対象者識別コードを付与する。研究対象者識別コードには、研究対象者の氏名等の研究対象者個人を特定することができる情報は用いない。本研究では、研究対象者の同定及び照会のため、研究対象者識別コードから研究対象者を特定することができる個人情報を管理する表（以下「対応表」という。）を作成するが、当該対応表は、作成した研究機関外への提供は一切行わない。

本研究結果が公表される場合にも、研究対象者個人を特定できる情報を含まないこととする。

また、本研究では研究対象者の写真や音声データを取得するが、本研究用にのみ用いるものとし、写真と音声データは研究対象者識別コードで管理を行う。（保管方法・廃棄方法は9.3、9.4項を参照。）

## 9.2. 試料／情報の授受

横浜市立大学 研究・産学連携推進センターでまとめられた情報やデータの解析結果は、委託元であるNTTデジタルツインコンピューティング研究センタと共有する。

症例登録票、中止報告書は、電子媒体で作成、保存し、下表に記載されたとおりに提出する。また本研究の情報収集はすべてWebアンケートで取得するため、症例報告書の作成は行わない。

ガーミンウォッチから得られるデータの取得方法については別添1にて説明する。

表1. 症例登録票の提出時期と提出方法

| 種類 | 提出時期 | 提出方法 | 提出先 |
| --- | --- | --- | --- |
| 症例登録票 | 症例登録時 | ファイルをパスワードで保護し、メール送付 | 登録センター（7項） |
| 中止報告書 | 事象発生時 | ファイルをパスワードで保護し、メール送付 | 研究事務局（23.4項） |

## 9.3. 試料・情報の保管方法及び保管期間

保管期間は少なくとも本研究の終了について報告された日から5年間もしくは当該研究の結果の最終の公表について報告された日から3年を経過した日のいずれか遅い日までの期間とするが、個人が特定できないよう加工した情報については二次利用の可能性があるため保管期間終了後も期間を定めず保管する）。（管理責任者：研究責任者）

保管方法は情報の種類に応じて以下のとおり管理する。

保管する情報の種類

・研究対象者の個人が特定できる情報を含む情報（対応表、同意書、同意撤回書、顔画像を含む画像データ、氏名が含まれる録音データ等

・研究対象者の個人が特定できないよう加工した情報

・研究に関する資料（研究計画書等の審査書類等）

それぞれ以下の通り保管する。

研究対象者の個人が特定できる情報について、電子データで情報を管理する場合は、研究・産学連携推進センターでネットワークから独立したパソコン/記録メディアを保管庫/部屋で施錠管理する。研究対象者の個人情報を含まない情報（Webアンケート等の個人が特定できないよう加工した情報、中止報告書）は、研究・産学連携推進センターで、パスワードで管理されたパソコン/USBメモリ等の記録媒体にて保管する。また対応表とは分けて管理することとする。また、同意書・同意撤回書については、紙媒体として原本保管するが、スキャンされた電子データは写しとして扱うものとする。

研究に関する資料（以下参照）は、立ち入りが管理された研究・産学連携推進センターで保管する：（1）研究計画書（2）その他倫理審査委員会へ提出した書類/承認された書類（3）倫理審査委員会の結果通知書および研究機関の長の実施許可書（4）その他本研究に関連する文書又は記録

破棄方法：保管期間を経過した文書および記録は、個人情報や機密情報の漏洩がないように細心の注意を払い廃棄する。コンピュータ上にある情報、記録メディアに記録されたデータを完全に削除する。

保管期間は、少なくとも研究の終了について報告された日から5年間もしくは当該研究の結果の最終の公表について報告された日から3年を経過した日のいずれか遅い日までの期間とするが、個人を特定できないよう加工されたデータについては、本研究の目的以外の学術研究に用いられる可能性または他の研究機関に提供する可能性（以下「二次利用」という。）があるため、保管期間終了後も期間を定めず保管する。保管期間を経過した文書および記録は、個人情報や機密情報の漏洩がないように細心の注意を払い廃棄する。コンピュータ上にある情報はデータ消去ソフトを使用するなどして完全に削除する。（管理責任者：研究責任者）

【登録センター】

収集・保管する情報：症例登録票（研究ID、適格性の結果）

管理方法：ID/PASSで管理されたクラウド上に保管を行う。

破棄方法：クラウド上にある情報は保管期間を経過した時点でデータ消去ソフトを使用するなどして完全に削除する。データの保管期間に関しては、研究の終了について報告された日から5年間とする。

【NTTデジタルツインコンピューティング研究センタ】

収集・保管する情報：ニックネーム、所在地、趣味（選択項目）、相手との関係、顔写真、音声データ

管理方法：研究対象者の個人が特定できる情報については、ネットワークから独立したパソコン/記録メディアを保管庫/部屋で施錠管理する。研究対象者の個人情報を含まない情報（Webアンケート等の個人が特定できないよう加工した情報）は、パスワードで管理されたパソコン/USBメモリ等の記録媒体にて保管する。

破棄方法：保管期間を経過した時点でデータ消去ソフトを使用するなどして完全に削除する。データの保管期間に関しては、研究の終了について報告された日から5年間とする。

## 9.4. 情報の廃棄方法

紙媒体で保管している資料は、シュレッダーにかけて廃棄する。電子データは、（記録メディアの破壊等）保存している端末から復元できない方法で消去する。

## 9.5. 情報の提供の記録

情報を提供の記録として、本研究計画書を保管する。

情報を提供する場合は提供した日から3年を経過した日まで、情報の提供を受ける場合は当該研究の終了について報告された日から5年を経過した日までの期間、適切に保管されるよう必要な監督を行う。

## 9.6. 情報の二次利用

本研究で収集した情報は、本研究の目的以外の学術研究に二次利用する可能性がある。二次利用の場合は、大学のホームページなどで情報を公開し、オプトアウトにて実施する。

# 10. 個人情報の保護

## 10.1. 個人情報の取扱い

　［遵守する法令、規定等］

1) 個人情報の管理者は、公立大学法人横浜市立大学人を対象とする生命科学・医学系研究に関する標準業務手順書に則り、研究責任者とする。
2) 本研究の関係者は、研究対象者の個人情報保護について個人情報保護法（平成15年法律第57号）を遵守する。ただし、個人情報のうち保有個人データの開示等に関して、個人情報保護法又は条例を遵守する。
3) 本研究の結果を学会や論文にて公表する際には、研究対象者の氏名等特定の個人を識別することができる情報を使用しない等、研究対象者の個人情報の保護について十分に配慮する。
4) 研究責任者および研究分担者は、所属する研究機関が保有する個人情報の漏えい、滅失又はき損の防止その他の安全管理措置等について、当該研究機関における安全管理措置に関する規定等に従い、適切に取り扱う。

　［個人情報の管理］

作成した対応表は、加工された個人情報と照合することにより特定の個人を識別できるため、当該資料を作成した研究機関外への提供は一切行わない。

## 10.2. 個人情報の開示等への対応

研究対象者本人等から、本研究により取得され、保有する個人情報のうち本人を識別することができるものについて、開示を求められた場合は、研究責任者又は研究分担者等が対応する。

## 10.3. 個人情報の取扱い等に関する問合せ及び苦情への対応

本研究における個人情報の取扱い等に関する問合せや苦情は、研究責任者又は研究事務局が対応する。

# 11. 研究結果（偶発的所見を含む）の取り扱い

## 11.1. 研究結果の説明方針

本研究では研究対象者の健康状態に影響を及ぼす情報を解析しない。そのため結果は開示しない。

## 11.2. 偶発的所見の取り扱い方針

本研究は、研究対象者の健康に重大な影響を及ぼすおそれのある所見が発見される可能性はない。

## 11.3. 研究対象者への説明方法

本研究では、研究結果の説明は行わず、偶発的所見が得られる可能性もないため、本項目は該当しない。

## 11.4. 遺伝カウンセリングの体制

本研究では遺伝子解析を行わないため、本項目は該当しない。

# 12. 研究対象者に生じる負担並びに予測されるリスク及び利益

## 12.1. 利益

本研究参加により研究対象者に直接の利益は生じない。

## 12.2. 負担・リスク及びそれらを最小化する対策

　本研究では、研究対象者に精神評価尺度の評価を行う。身体的負担・リスクはないが、回答するため約10分の時間的拘束が生じる（動画の視聴から対話後のアンケート調査までの時間に含まれる）。また、質問内容に不安や恐怖、抑うつ症状が含まれており、心理的負担が生じる可能性がある。研究参加の同意を得る時点で、心理的負担が生じる可能性があること、回答したくない質問には回答しなくてよいことを予め説明し、心理的負担の軽減に努める。また、研究参加に同意した後（たとえ研究期間中であっても）、いつでも同意を撤回することができることを説明する。精神的な問題が生じることは考えにくいが、問題が生じた場合の対応として、所属大学や所属企業の保健管理センター／健康管理室への相談勧奨や近医への受診勧奨を行う。

　また、動画の視聴から対話後のアンケート調査までで約60分間の時間的拘束が生じる。なお、動画の視聴に関しては通信料が発生するが、研究対象者の負担とする。

# 13. 健康被害に対する補償

本研究は情報のみを用いる研究であり、健康被害が生じる可能性はないため、補償は準備しない。

# 14. 研究対象者の費用負担・謝礼

　本研究に参加する研究対象者へ、交通費として2000円＋謝礼として5000円分のAmazonギフトを研究終了時に支払う。なお、研究途中で脱落した者に関しては、交通費の支払いは発生するが謝礼の支払いは発生しない。

# 15. 研究の資金源

本研究は、NTTデジタルコンピューティング研究センタからの委託契約により実施する臨床研究である。

# 16. 利益相反

本研究は、NTTデジタルコンピューティング研究センタより研究資金の提供を受ける。本研究の利害関係については、横浜市立大学臨床研究利益相反委員会の意見を聴き、適切に利益相反マネジメントを行う。また、本研究の進捗状況等に応じて適宜、横浜市立大学臨床研究利益相反委員会へ報告を行い、本研究の利害関係についての公正性を保つものとし、研究成果公開時には当該利益相反の事実について開示する。

# 17. 研究実施にかかる手続

## 17.1. 遵守すべき諸規則

本研究は「ヘルシンキ宣言」および「人を対象とする生命科学・医学系研究に関する倫理指針」を参照して実施する。

## 17.2. 倫理審査委員会への付議

研究責任者は、本研究を実施する前に倫理審査委員会による審査を受け、承認を得る。また、研究計画書等の変更又は改訂を行う場合にも、定められた手順に従って倫理審査委員会による審査を受け、承認を得る。

## 17.3. 研究機関の長による許可等

研究責任者は倫理審査委員会の審査の審査結果、審査過程のわかる記録及び当該倫理審査委員会の委員の出欠状況、承認された資料をもって、研究機関の長により研究の実施許可を得る。初回の審査に限らず、倫理審査委員会の審査を受けた際にはその都度研究機関の長の許可を得るものとする。

## 17.4. 定期報告

研究責任者は、本研究の実施状況を倫理審査委員会の求めに応じて倫理審査委員会及び研究機関の長に報告する。

## 17.5. 研究の停止・中止

研究者等は、研究の継続に影響を与えると考えられる情報を得た場合、当該機関の研究責任者に報告し、研究責任者は研究機関の長へ報告する。

研究責任者は必要に応じて研究を停止若しくは中止し、又は研究計画書の変更を行う。研究機関の長より研究の停止又は中止の指示を受けた場合は、速やかに対応する。

## 17.6. 研究の終了

研究責任者は、目標対象者数の予定した解析が終了した場合、又は得られた情報が研究目的を検討するのに十分なため早期に終了する場合、研究が終了した旨及び研究の結果の概要を遅滞なく倫理審査委員会及び研究機関の長に報告する。

# 18. モニタリング及び監査

　　本研究は観察研究であるため、モニタリング及び監査は行わない。

# 19. 研究の情報公開及び結果の公表

本研究の研究概要及び研究結果は国立大学附属病院長会議他が設置している公開データベース（UMIN＝University Hospital Medical Information Network）に登録し、研究進捗に応じて適宜情報の更新等を行う。研究終了後、本研究から得られた成果を国内外の学会にて発表、論文化により公表する。

# 20. 研究成果の帰属

研究成果は原則として横浜市立大学に帰属するが、委託元であるNTTデジタルツインコンピューティング研究センタから開示された情報に基づいてなした場合の成果は、共有に帰属する。

# 21. 研究対象者及びその関係者からの相談等への対応

研究事務局が対応する。（23.3.項参照）

# 22. 研究に関する業務の委託

本研究に関する対象者登録業務は、株式会社ヌーベルプラスに委託し、指名された担当者が実施する。委託業務の実施状況等は委受託契約書に基づいて監督する。

機関名：株式会社ヌーベルプラス

住所：東京都港区虎ノ門3-8-21

委託する業務の内容：症例登録業務

責任者：吉田　裕彦

監督責任者：研究責任者

# 23. 研究実施体制

## 23.1. 研究責任者

機関名：横浜市立大学

所属：研究・産学連携推進センター

氏名：宮﨑　智之

住所: 〒220-0012 横浜市西区みなとみらい3-7-1

オーシャンゲートみなとみらい8F

TEL：050-3575-7535

## 23.2. 既存試料・情報の提供のみを行う機関と責任者

機関名：NTTデジタルツインコンピューティング研究センタ

所属：日本電信電話株式会社 人間情報研究所

責任者氏名：深山 篤

電話：03-6810-4016

## 23.3. 研究事務局

機関名：横浜市立大学

所属：研究・産学連携推進センター

氏名：山里　亜未・謝　雪こう

電話：050-3575-7535

E-mail：takeda.ami.nm@yokohama-cu.ac.jp

direnjie3322@hotmail.com

役割：研究対象者及びその関係者からの相談対応、各種事務手続きを行う

## 23.4. 統計解析責任者

機関名：横浜市立大学

所属：研究・産学連携推進センター

氏　名　：宮﨑　智之

電話：050-3575-7535

## 23.5. データセンター及びマネジメント責任者

機関名：横浜市立大学

所属：研究・産学連携推進センター

氏　名　：宮﨑　智之

電話：050-3575-7535

# 24. 引用文献

1) Jones, W. H., & Russell, D.1982 The social reticence scale. An objective instrument to measure shyness. Journal of Personality Assessment, 46, 629-631.
2) 野村竜也（2022）「対人不安とHuman-Robot Interaction」　『計測と制御』61-(3)
3) American Psychiatric Association(2013): Diagnostic and statisti- cal manual of mental disorders, 5th ed., Washington, D. C.: American Psychiatric Association
4) 福田正人、寺崎正治（2012）「シャイネスが日常活動および主観的幸福感に及ぼす影響」『川崎医療福祉学会誌』21-(2)
5) 厚生労働省(2007)ニートの状態にある若年者の実態及び支援策に関する調査研究[http://www.mhlw.go.jp/houdou/200 7/06/h0628-1.html](http://www.mhlw.go.jp/houdou/200%207/06/h0628-1.html) (2023年11月6日)
6) 安井梨恵・米山直樹(2010)「シャイネス に対する認知行動療法的アプローチに関する考察」『人文論究』60(1)
7) Leary,M.R(1986) Attective and behavioral components of shyness: Implications for theory, measurement, and research. In W.H.Jones, J.M Cheek and S.R. Brigga(Eds.),Shyness: Perspeectives on research dan treatment.. New York :Plenum Press.
8) 西村洋一(2002)「対人不安,インターネット利用，およびインターネットにおける人間関係」『社会心理学研究』19–2
9) 杉谷陽子(2007)「メールはなぜ「話しやすい」のか？CMC(Computer-Mediated Communication)における自己呈示効力感の上昇」『社会心理学研究』22–3
10) https://www.univcoop.or.jp/about/campus_life/vol63_02.html
11) Gresham, F.M.(1988) Social skills : Conceptual and applied aspects of assessment, training, and social validation, In Witt, J.C., Elliott, S.N. & Gresham, F.M.(Eds.), Handbook of behavior therapy in education. New York Press.pp.
12) 徳永沙智、稲畑陽子、原田素美礼、境泉洋（2013）「シャイネスと被受容感・被拒絶感が社会的スキルに及ぼす影響」『徳島大学 人間科学研究』21

別添

データ取得の手順書

ガーミンウォッチから得られるデータは、ユーザーのデバイスにインストールされた専用のアプリを通じて集められ、一時的にAWS（Amazon Web Services）のデータサーバーに保存される。そして、適宜、API接続を通じて、これらのデータは横浜市立大学のデータサーバーに転送される。対象者が使用しているガーミンデバイスID、生体データが横浜市立大学のサーバーに転送される。
