# Supplementary material for: Investigating “Another Me” digital twin technology to support the development of human relationships: An exploratory randomized controlled study
Source: PLoS One. 2026 Aug 7;21(8):e0353835. doi: 10.1371/journal.pone.0353835 (PMC13450786; doi:10.1371/journal.pone.0353835)
Supplement: Supplementary file 6 [file pone.0353835.s009.docx]

**Response to the Academic Editor and Reviewers**

We sincerely thank the Academic Editor and the Reviewers for their constructive suggestions. We have carefully revised the manuscript in accordance with all suggestions, where all changes have been incorporated into the revised version.

**Response to Reviewer #1**

We are grateful for the positive overall evaluation of our exploratory randomized trial. Your constructive suggestions help us to enhance interpretability and transparency of our manuscript.

Comment 1: The primary endpoint was not statistically significant. Please align the Abstract and Conclusion with the primary analysis and clearly frame time-course findings as exploratory.

We appreciate your important comment and have revised the Abstract, Discussion, and Conclusion accordingly.

1. We explicitly state in the Abstract and Discussion that the primary endpoint was not statistically significant (P.2, L36-38; P15, L324-326).
2. We clarified the distinction between primary and exploratory secondary analyses (P.2, L38-40; P.8, L198-200, L203-204).
3. We explicitly declared the time-course findings as exploratory (P.2, L38-40).
4. We revised the Abstract and Conclusion to avoid overinterpretation (P.2, L42-43; P.19, L436-440).

Comment 2: Clarify that the estimand is the pre-dialogue simulation viewing procedure using Another Me, rather than the isolated effect of Another Me itself.

We agree that the observed group differences should be derived by a multi-component pre-dialogue simulation viewing procedure rather than the isolated effect of the digital twin technology itself. To clarify this, we revised corresponding sentences accordingly.

1. We added the following sentence to the Abstract (P.14, L313-316):

“The results of this study suggest that the pre-dialogue simulation viewing procedure using Another Me digital twin technology may reduce pre-conversation psychophysiological burden and subjective anxiety, rather than demonstrating the isolated effect of Another Me technology itself.”

2. We revised the Conclusion to clarify this point (P.19, L441 L444).

Comment 3: The change in primary measurement method should also be acknowledged in the Limitations.

We have added this issue as the sixth limitation in Discussion (P.18, L427- P.19, L432). Specifically, we here state clearly that the primary measurement method was changed during the study from continuous heart rate monitoring using a smartwatch to pulse rate measurement using an upper-arm blood pressure monitor because of technical issues. Although this change was approved by the ethics committee prior to data analysis, the use of intermittent pulse measurements may have reduced sensitivity for detecting transient physiological changes and may have weakened the evidentiary strength of the primary endpoint.

Comment 4: Clarify the rule for interpreting secondary analyses and consider sensitivity analysis controlling the false discovery rate.

We appreciate this suggestion. In our study, formal hypothesis testing was conducted only for the primary endpoint. For secondary endpoints and time-course summaries, we reported means and 95% confidence intervals without conducting inferential statistical tests (This analysis policy was prespecified in our Statistical Analysis Plan). Therefore, issues of multiplicity did not arise, and adjustment for the false discovery rate was not applicable.

However, we identified an inconsistency in the original Statistical Analysis section and have corrected it for full transparency (P.8, L198-202).

In addition, to avoid ambiguity, we have clearly distinguished and reorganized the descriptions of (1) the main analysis of the primary endpoint, (2) secondary time-course analyses, and (3) secondary endpoints. This restructuring clarifies which analyses involved formal hypothesis testing and which were descriptive and hypothesis-generating (P.8, L198-205).

We also corrected an inaccurate description in the Discussion to ensure consistency with the revised Statistical Analysis section (P.15, L319-322).

**Response to Reviewer #2**

We sincerely thank the reviewer for highlighting the importance of digital twin validity and fidelity.

Comment1: You mentioned the importance of validation and verification procedures as well as continuous assessment of the model’s fidelity over time, and you asked us to add these points in discussion and limitation.

We agree that validation and fidelity assessment are critical when applying digital twin technology, particularly with respect to whether the digital twin representation accurately reflects the individual.

To address this concern:

1. We added the Player Identification Scale (PIS), a measure of avatar identification, to the Psychological Data section in the Methods (P.7, L180-P.8, L184).
2. We included the results of the PIS in the Secondary Endpoints section of the Results (P.13, L.293- P14, L295).
3. In the Discussion, we incorporated evidence from previous studies demonstrating the reproducibility of Another Me prior to this study. In this study we firstly adopted the PIS findings to show the evidence describing the fidelity of Another Me. Based on these results, we clarified that Another Me appears to achieve a certain degree of person-specific fidelity and subjective identification. (P.17, L395- P.18, L408)
4. In the Limitations section, we explicitly stated that we did not perform an end-to-end validation of the integrated Another Me system against each participant’s actual conversational behavior in matched interactional settings (P.18, L422-424).

Comment2: State the impact on results due to the change from a smartwatch to a blood pressure monitor in your analysis.

As noted above in responding to Reviewer#1, we have added the sentence describing our change regarding measurement device to the Limitations section and clarified its potential impact on sensitivity and evidentiary strength (P.18, L427-432).

We sincerely appreciate the reviewers’ constructive feedback, which significantly improved the clarity, transparency, and scientific rigor of our manuscript. We hope that the revised version adequately addresses all concerns and we respectfully submit it for reconsideration.

Thank you very much for your time and consideration.
