# Supplementary material for: Investigating “Another Me” digital twin technology to support the development of human relationships: An exploratory randomized controlled study
Source: PLoS One. 2026 Aug 7;21(8):e0353835. doi: 10.1371/journal.pone.0353835 (PMC13450786; doi:10.1371/journal.pone.0353835)
Supplement: Peer review history

# Peer review: Decision Letter 0

**DOI:** 10.1371/journal.pone.0353835.r001

**Authors:** Yoshitaka Ishibashi

12 Feb 2026

PONE-D-25-59383

Investigating “Another Me” digital twin technology to support the development of human relationships: An exploratory randomized controlled study

PLOS One

Dear Dr. Miyazaki,

Thank you for submitting your manuscript to PLOS ONE. After careful consideration, we feel that it has merit but does not fully meet PLOS ONE’s publication criteria as it currently stands. Therefore, we invite you to submit a revised version of the manuscript that addresses the points raised during the review process.

Please submit your revised manuscript by Mar 28 2026 11:59PM. If you will need more time than this to complete your revisions, please reply to this message or contact the journal office at plosone@plos.org. When you're ready to submit your revision, log on to https://www.editorialmanager.com/pone/ and select the 'Submissions Needing Revision' folder to locate your manuscript file.

Please include the following items when submitting your revised manuscript:

- A letter that responds to each point raised by the academic editor and reviewer(s). You should upload this letter as a separate file labeled 'Response to Reviewers'.
- A marked-up copy of your manuscript that highlights changes made to the original version. You should upload this as a separate file labeled 'Revised Manuscript with Track Changes'.
- An unmarked version of your revised paper without tracked changes. You should upload this as a separate file labeled 'Manuscript'.

If you would like to make changes to your financial disclosure, please include your updated statement in your cover letter. Guidelines for resubmitting your figure files are available below the reviewer comments at the end of this letter.

If applicable, we recommend that you deposit your laboratory protocols in protocols.io to enhance the reproducibility of your results. Protocols.io assigns your protocol its own identifier (DOI) so that it can be cited independently in the future. For instructions see: https://journals.plos.org/plosone/s/submission-guidelines#loc-laboratory-protocols. Additionally, PLOS ONE offers an option for publishing peer-reviewed Lab Protocol articles, which describe protocols hosted on protocols.io. Read more information on sharing protocols at https://plos.org/protocols?utm_medium=editorial-email&utm_source=authorletters&utm_campaign=protocols.

We look forward to receiving your revised manuscript.

Kind regards,

Yoshitaka Ishibashi

Academic Editor

PLOS One

Journal Requirements:

When submitting your revision, we need you to address these additional requirements.

1. Please ensure that your manuscript meets PLOS ONE's style requirements, including those for file naming. The PLOS ONE style templates can be found at

https://journals.plos.org/plosone/s/file?id=wjVg/PLOSOne_formatting_sample_main_body.pdf and https://journals.plos.org/plosone/s/file?id=ba62/PLOSOne_formatting_sample_title_authors_affiliations.pdf

2. Thank you for stating in your Funding Statement:

[This project was supported by JST Grant Number J JPMJPF2203 (T.M.) and the NTT Digital Computing Research Center (T.M.). The authors declare that this company was not involved in the statistical analysis and interpretation of the results.].

Please provide an amended statement that declares *all* the funding or sources of support (whether external or internal to your organization) received during this study, as detailed online in our guide for authors at http://journals.plos.org/plosone/s/submit-now.  Please also include the statement “There was no additional external funding received for this study.” in your updated Funding Statement.

Please include your amended Funding Statement within your cover letter. We will change the online submission form on your behalf.

3. Please expand the acronyms “NTT” and “JST” (as indicated in your financial disclosure) so that it states the name of your funders in full.

This information should be included in your cover letter; we will change the online submission form on your behalf.

4. Thank you for stating the following in the Competing Interests section:

[The authors declare that they received financial support as well as technical support about Another Me from the NTT Digital Computing Research Center, which was not involved in the statistical analysis and interpretation of the results.].

Please confirm that this does not alter your adherence to all PLOS ONE policies on sharing data and materials, by including the following statement: "This does not alter our adherence to PLOS ONE policies on sharing data and materials.” (as detailed online in our guide for authors http://journals.plos.org/plosone/s/competing-interests).  If there are restrictions on sharing of data and/or materials, please state these. Please note that we cannot proceed with consideration of your article until this information has been declared.

Please include your updated Competing Interests statement in your cover letter; we will change the online submission form on your behalf.

5. Thank you for stating the following in the Competing Interests section:

[The authors declare that they received financial support as well as technical support about Another Me from the NTT Digital Computing Research Center, which was not involved in the statistical analysis and interpretation of the results.].

We note that one or more of the authors are employed by a commercial company: NTT Inc.

1. Please provide an amended Funding Statement declaring this commercial affiliation, as well as a statement regarding the Role of Funders in your study. If the funding organization did not play a role in the study design, data collection and analysis, decision to publish, or preparation of the manuscript and only provided financial support in the form of authors' salaries and/or research materials, please review your statements relating to the author contributions, and ensure you have specifically and accurately indicated the role(s) that these authors had in your study. You can update author roles in the Author Contributions section of the online submission form.

Please also include the following statement within your amended Funding Statement.

“The funder provided support in the form of salaries for authors [insert relevant initials], but did not have any additional role in the study design, data collection and analysis, decision to publish, or preparation of the manuscript. The specific roles of these authors are articulated in the ‘author contributions’ section.”

If your commercial affiliation did play a role in your study, please state and explain this role within your updated Funding Statement.

2. Please also provide an updated Competing Interests Statement declaring this commercial affiliation along with any other relevant declarations relating to employment, consultancy, patents, products in development, or marketed products, etc.

Within your Competing Interests Statement, please confirm that this commercial affiliation does not alter your adherence to all PLOS ONE policies on sharing data and materials by including the following statement: "This does not alter our adherence to PLOS ONE policies on sharing data and materials.” (as detailed online in our guide for authors http://journals.plos.org/plosone/s/competing-interests) . If this adherence statement is not accurate and there are restrictions on sharing of data and/or materials, please state these. Please note that we cannot proceed with consideration of your article until this information has been declared.

Please include both an updated Funding Statement and Competing Interests Statement in your cover letter. We will change the online submission form on your behalf.

6. In the online submission form you indicate that your data is not available for proprietary reasons and have provided a contact point for accessing this data. Please note that your current contact point is a co-author on this manuscript. According to our Data Policy, the contact point must not be an author on the manuscript and must be an institutional contact, ideally not an individual. Please revise your data statement to a non-author institutional point of contact, such as a data access or ethics committee, and send this to us via return email. Please also include contact information for the third-party organization, and please include the full citation of where the data can be found.

7. Please include a separate caption for each figure in your manuscript.

8. We note that S1 Text includes an image of a participant in the study.

As per the PLOS ONE policy (http://journals.plos.org/plosone/s/submission-guidelines#loc-human-subjects-research) on papers that include identifying, or potentially identifying, information, the individual(s) or parent(s)/guardian(s) must be informed of the terms of the PLOS open-access (CC-BY) license and provide specific permission for publication of these details under the terms of this license. Please download the Consent Form for Publication in a PLOS Journal (http://journals.plos.org/plosone/s/file?id=8ce6/plos-consent-form-english.pdf). The signed consent form should not be submitted with the manuscript, but should be securely filed in the individual's case notes. Please amend the methods section and ethics statement of the manuscript to explicitly state that the patient/participant has provided consent for publication: “The individual in this manuscript has given written informed consent (as outlined in PLOS consent form) to publish these case details”.

If you are unable to obtain consent from the subject of the photograph, you will need to remove the figure and any other textual identifying information or case descriptions for this individual.

9. We note that S1 Text in your submission contains copyrighted images. All PLOS content is published under the Creative Commons Attribution License (CC BY 4.0), which means that the manuscript, images, and Supporting Information files will be freely available online, and any third party is permitted to access, download, copy, distribute, and use these materials in any way, even commercially, with proper attribution. For more information, see our copyright guidelines: http://journals.plos.org/plosone/s/licenses-and-copyright.

We require you to either (1) present written permission from the copyright holder to publish these figures specifically under the CC BY 4.0 license, or (2) remove the figures from your submission:

1. You may seek permission from the original copyright holder of S1 Text to publish the content specifically under the CC BY 4.0 license.

We recommend that you contact the original copyright holder with the Content Permission Form (http://journals.plos.org/plosone/s/file?id=7c09/content-permission-form.pdf) and the following text:

“I request permission for the open-access journal PLOS ONE to publish XXX under the Creative Commons Attribution License (CCAL) CC BY 4.0 (http://creativecommons.org/licenses/by/4.0/). Please be aware that this license allows unrestricted use and distribution, even commercially, by third parties. Please reply and provide explicit written permission to publish XXX under a CC BY license and complete the attached form.”

Please upload the completed Content Permission Form or other proof of granted permissions as an "Other" file with your submission.

In the figure caption of the copyrighted figure, please include the following text: “Reprinted from [ref] under a CC BY license, with permission from [name of publisher], original copyright [original copyright year].”

2. If you are unable to obtain permission from the original copyright holder to publish these figures under the CC BY 4.0 license or if the copyright holder’s requirements are incompatible with the CC BY 4.0 license, please either i) remove the figure or ii) supply a replacement figure that complies with the CC BY 4.0 license. Please check copyright information on all replacement figures and update the figure caption with source information. If applicable, please specify in the figure caption text when a figure is similar but not identical to the original image and is therefore for illustrative purposes only.

10. We note that there is identifying data in the Supporting Information file <S1 Text. Creating a Digital Twin.docx>. Due to the inclusion of these potentially identifying data, we have removed this file from your file inventory. Prior to sharing human research participant data, authors should consult with an ethics committee to ensure data are shared in accordance with participant consent and all applicable local laws.

Data sharing should never compromise participant privacy. It is therefore not appropriate to publicly share personally identifiable data on human research participants. The following are examples of data that should not be shared:

-Name, initials, physical address

-Ages more specific than whole numbers

-Internet protocol (IP) address

-Specific dates (birth dates, death dates, examination dates, etc.)

-Contact information such as phone number or email address

-Location data

-ID numbers that seem specific (long numbers, include initials, titled “Hospital ID”) rather than random (small numbers in numerical order)

Data that are not directly identifying may also be inappropriate to share, as in combination they can become identifying. For example, data collected from a small group of participants, vulnerable populations, or private groups should not be shared if they involve indirect identifiers (such as sex, ethnicity, location, etc.) that may risk the identification of study participants.

Additional guidance on preparing raw data for publication can be found in our Data Policy (https://journals.plos.org/plosone/s/data-availability#loc-human-research-participant-data-and-other-sensitive-data) and in the following article: http://www.bmj.com/content/340/bmj.c181.long.

Please remove or anonymize all personal information (name, age, location data), ensure that the data shared are in accordance with participant consent, and re-upload a fully anonymized data set. Please note that spreadsheet columns with personal information must be removed and not hidden as all hidden columns will appear in the published file.

11. We note that the original protocol file you uploaded contains a confidentiality notice indicating that the protocol may not be shared publicly or be published. Please note, however, that the PLOS Editorial Policy requires that the original protocol be published alongside your manuscript in the event of acceptance. Please note that should your paper be accepted, all content including the protocol will be published under the Creative Commons Attribution (CC BY) 4.0 license, which means that it will be freely available online, and any third party is permitted to access, download, copy, distribute, and use these materials in any way, even commercially, with proper attribution.

Therefore, we ask that you please seek permission from the study sponsor or body imposing the restriction on sharing this document to publish this protocol under CC BY 4.0 if your work is accepted. We kindly ask that you upload a formal statement signed by an institutional representative clarifying whether you will be able to comply with this policy. Additionally, please upload a clean copy of the protocol with the confidentiality notice (and any copyrighted institutional logos or signatures) removed.

If the reviewer comments include a recommendation to cite specific previously published works, please review and evaluate these publications to determine whether they are relevant and should be cited. There is no requirement to cite these works unless the editor has indicated otherwise.

Additional Editor Comments:

Dear Dr. Miyazaki,

Thank you for submitting your manuscript entitled

**“Investigating ‘Another Me’ digital twin technology to support the development of human relationships: An exploratory randomized controlled study”**  to *PLOS ONE* .

The manuscript has now been evaluated by a reviewer with relevant expertise. The reviewer finds that the study is carefully designed, clearly reported, and of high scientific quality for an exploratory randomized controlled trial. The use of digital twin technology to reduce the psychological burden of first-time social interactions is considered novel and potentially impactful.

However, to improve interpretability, transparency, and methodological clarity, **minor revisions**  are required before the manuscript can be considered for acceptance. These revisions do not call into question the overall validity of the study, but rather aim to ensure that conclusions are appropriately aligned with the study design and statistical evidence.

Please address the reviewers comments in a revised version of the manuscript:

We believe that addressing these points will further strengthen the manuscript and enhance its transparency and contribution to the field. We therefore invite you to submit a revised manuscript that carefully responds to the reviewer’s comments.

We look forward to receiving your revised submission.

Sincerely,

Yoshitaka Ishibashi

[Note: HTML markup is below. Please do not edit.]

Reviewers' comments:

Reviewer's Responses to Questions

**Comments to the Author**

1. Is the manuscript technically sound, and do the data support the conclusions?

The manuscript must describe a technically sound piece of scientific research with data that supports the conclusions. Experiments must have been conducted rigorously, with appropriate controls, replication, and sample sizes. The conclusions must be drawn appropriately based on the data presented.

Reviewer #1: Yes

Reviewer #2: Yes

**********

2. Has the statistical analysis been performed appropriately and rigorously?

Reviewer #1: Yes

Reviewer #2: Yes

**********

3. Have the authors made all data underlying the findings in their manuscript fully available?

The PLOS Data policy requires authors to make all data underlying the findings described in their manuscript fully available without restriction, with rare exception (please refer to the Data Availability Statement in the manuscript PDF file). The data should be provided as part of the manuscript or its supporting information, or deposited to a public repository. For example, in addition to summary statistics, the data points behind means, medians and variance measures should be available. If there are restrictions on publicly sharing data—e.g. participant privacy or use of data from a third party—those must be specified.

Reviewer #1: Yes

Reviewer #2: Yes

**********

4. Is the manuscript presented in an intelligible fashion and written in standard English?

PLOS ONE does not copyedit accepted manuscripts, so the language in submitted articles must be clear, correct, and unambiguous. Any typographical or grammatical errors should be corrected at revision, so please note any specific errors here.

Reviewer #1: Yes

Reviewer #2: Yes

**********

5. Review Comments to the Author

Please use the space provided to explain your answers to the questions above. You may also include additional comments for the author, including concerns about dual publication, research ethics, or publication ethics. (Please upload your review as an attachment if it exceeds 20,000 characters)

Reviewer #1: Overall Comment: This manuscript reports an exploratory randomized open-label controlled trial evaluating whether viewing a pre-dialogue simulation video generated using Another Me digital twin technology can reduce psychophysiological burden during a subsequent first-time online one-on-one conversation with a stranger. The study is carefully designed and clearly reported, and the work reaches a high scientific standard for an exploratory trial. To further improve interpretability and transparency, I recommend the following four revisions.

Comment 1: The primary endpoint, the between-group difference in heart rate change from before to after the online conversation, was not statistically significant. However, the Abstract and Conclusion emphasize a sustained reduction in heart rate through the end of the conversation based mainly on secondary time-course summaries. Please align the Abstract and Conclusion with the primary analysis, stating that the primary endpoint was not met and that secondary analyses suggest a trend toward lower pre-conversation heart rate and lower subjective anxiety and tension. Please also explicitly frame the time-course findings as exploratory.

Comment 2: The observed group differences are best interpreted as the effect of a procedure that uses Another Me for pre-dialogue simulation viewing rather than as the isolated effect of Another Me itself. Several components differ between groups, including avatar selection being performed only in the intervention group, and the intervention group conversing with the same interlocutor who appeared in the pre-viewed video whereas the control group conversed with an interlocutor who did not appear in the video. Please add this as a limitation and adjust wording throughout, especially in the Abstract and Conclusion, to describe the estimand as the pre-dialogue simulation viewing procedure using Another Me.

Comment 3: The change in the primary measurement approach from continuous heart rate monitoring with a smartwatch to pulse rate measured with an upper-arm blood pressure monitor is described in the Methods, but it should also be acknowledged in the Limitations because it directly affects the evidentiary strength of the primary endpoint.

Comment 4: Given the number of secondary outcomes and time points, it would strengthen the manuscript to state a clear rule for interpreting secondary analyses. Please consider adding a sensitivity analysis that controls the false discovery rate and report these results in the Supplementary Information, so readers can distinguish findings that are robust from those that should be treated as preliminary trends.

Reviewer #2: The approach of using digital twin technology (“Another Me”) to represent participants indirectly without exposing themselves is novel. The original aspect lies in reducing psychological burden by enabling smooth conversation reproduction in a digital environment.

When discussing the validity of reproducibility in generating an “Another Me,” it is important to consider whether the digital twin accurately and reliably represents the real individual. In the context of digital twin technology, the model must maintain equivalence between the digital representation and the physical source to ensure that generated behaviors or responses are meaningful and trustworthy for their intended use. This involves validation and verification procedures, as well as continuous assessment of the model’s fidelity over time. Without such validation, there is a risk that the reproduced “Another Me” may diverge from the real characteristics it is intended to reflect, undermining its credibility and usefulness.

Please add these points in discussion and limitation, moreover state the impact on results due to the change from a smartwatch to a blood pressure monitor in your analysis.

**********

6. PLOS authors have the option to publish the peer review history of their article (what does this mean?). If published, this will include your full peer review and any attached files.

If you choose “no”, your identity will remain anonymous but your review may still be made public.

**Do you want your identity to be public for this peer review?**  For information about this choice, including consent withdrawal, please see our Privacy Policy.

Reviewer #1: No

Reviewer #2: No

**********

[NOTE: If reviewer comments were submitted as an attachment file, they will be attached to this email and accessible via the submission site. Please log into your account, locate the manuscript record, and check for the action link "View Attachments". If this link does not appear, there are no attachment files.]

To ensure your figures meet our technical requirements, please review our figure guidelines: https://journals.plos.org/plosone/s/figures

You may also use PLOS’s free figure tool, NAAS, to help you prepare publication quality figures: https://journals.plos.org/plosone/s/figures#loc-tools-for-figure-preparation.

NAAS will assess whether your figures meet our technical requirements by comparing each figure against our figure specifications.

# Author comment: Author response to Decision Letter 1

**DOI:** 10.1371/journal.pone.0353835.r002

27 Mar 2026

Dear Dr. Ishibashi,

Thank you very much for your detailed comments and guidance. We have carefully addressed each of the points raised, as outlined below.

Comment 1: Please ensure that your manuscript meets PLOS ONE's style requirements, including those for file naming.

We have reviewed the PLOS ONE style templates and ensured that the manuscript complies with the required formatting.

Comment 2: Please provide an amended statement that declares *all* the funding or sources of support (whether external or internal to your organization) received during this study. Please also include the statement “There was no additional external funding received for this study.” in your updated Funding Statement. Please include your amended Funding Statement within your cover letter.

Comment 3: Please expand the acronyms “NTT” and “JST” (as indicated in your financial disclosure) so that it states the name of your funders in full.

Comment 4: Please confirm that this does not alter your adherence to all PLOS ONE policies on sharing data and materials, by including the following statement: "This does not alter our adherence to PLOS ONE policies on sharing data and materials.”

We have revised and included the Funding Statement and Competing Interests Statement in the cover letter, as requested.

Comment 5-1: We note that one or more of the authors are employed by a commercial company: NTT Inc.

Please provide an amended Funding Statement declaring this commercial affiliation, as well as a statement regarding the Role of Funders in your study. Please also include the specified statement.

We have revised the Funding Statement to clearly declare the commercial affiliation of several authors with NTT Inc. In addition, we have clarified the role of the funders in the study.

Specifically:

We explicitly stated that multiple authors are employees of NTT Inc.

We clarified that NTT provided research funding (not salary-based support specific to this study).

Comment 5-2: Please also provide an updated Competing Interests Statement declaring this commercial affiliation along with any other relevant declarations. Please confirm that this does not alter adherence to PLOS ONE policies on sharing data and materials.

We have revised the Competing Interests Statement to explicitly declare the employment relationships with NTT Inc.

In addition:

We confirmed that this commercial affiliation does not affect compliance with PLOS ONE policies.

We included the required statement regarding adherence to data and materials sharing policies.

Comment 6: In the online submission form you indicate that your data is not available for proprietary reasons and have provided a contact point for accessing this data. Please note that your current contact point is a co-author on this manuscript. According to our Data Policy, the contact point must not be an author on the manuscript and must be an institutional contact, ideally not an individual. Please revise your data statement accordingly and provide contact information for the third-party organization.

In accordance with the journal’s Data Policy, we have revised the data statement to designate a non-author institutional contact point for data access. The contact point is now an appropriate third-party institutional office, rather than an individual author.

The contact point for accessing the data has been updated as follows:

Research Team Administration

3-7-1 Minatomirai, Nishi-ku, Yokohama, Kanagawa, Japan

Tel: +81-50-3575-7535

Email: coinext@yokohama-cu.ac.jp

Comment 7: Please include a separate caption for each figure in your manuscript.

In accordance with the PLOS ONE style template, we have revised the manuscript so that all figure captions are included within the main text, placed immediately after the paragraphs in which the figures are first cited.

Comment 8: We note that S1 Text includes an image of a participant in the study. As per the PLOS ONE policy on identifying information, please ensure that appropriate written informed consent for publication under the CC-BY license has been obtained, and amend the Methods and Ethics Statement accordingly.

The facial images included are not of study participants but of co-authors.

To avoid any misunderstanding, we have explicitly stated this in the figure captions.

In addition, we have taken the following steps to ensure compliance with PLOS ONE policy:

We obtained written informed consent for publication using the Consent Form for Publication in a PLOS Journal from the individuals shown in the images.

We have securely retained the signed consent forms as required by the journal policy.

We revised the Methods section (Ethics Statement) to explicitly indicate that consent for publication has been obtained.

The following statement has been added to the manuscript:

“The individuals shown in this figure 6, 7 are co-authors and have given written informed consent (as outlined in the PLOS consent form) to publish these images.”

Comment 9: We note that S1 Text in your submission contains copyrighted images. Please provide permission from the copyright holder for publication under the CC BY 4.0 license, or remove/replace the figures accordingly.

The image included in S1 Text is an original photograph taken by the authors and is not reproduced from any copyrighted source; therefore, permission from a third-party copyright holder is not required. The individual shown is a co-author, and written informed consent for publication has been obtained in accordance with the PLOS consent form.

To ensure clarity and avoid any misunderstanding, we have revised the figure legend in S1 Text to explicitly state that:

・the image is original and created by the authors, and

・the individual depicted is a co-author who has provided written informed consent for publication.

Comment 10: We note that there is identifying data in the Supporting Information file <S1 Text. Creating a Digital Twin.docx>. Please remove or anonymize all personal information and re-upload a fully anonymized dataset in accordance with participant consent and applicable policies.

In response to this comment, we have carefully reviewed the Supporting Information file and revised it to ensure that all potentially identifying information has been appropriately anonymized.

Initials used in the file have been replaced with anonymized labels (e.g., “Person A”, “Person B”) to prevent identification of individuals.

The revised, fully anonymized Supporting Information file has been re-uploaded.

Comment 11: We note that the original protocol file contains a confidentiality notice indicating that it may not be shared publicly. As PLOS requires that the protocol be published under a CC BY 4.0 license upon acceptance, please obtain permission from the relevant authority and provide a formal statement. Additionally, please upload a clean copy of the protocol with the confidentiality notice removed.

Regarding the protocol sharing requirements, we have obtained institutional approval for making the study protocol publicly available. A formal statement signed by an institutional representative has been uploaded.

In addition, we have uploaded a clean version of the protocol with the confidentiality notice and any elements incompatible with open-access publication removed.

We hope that these revisions meet the journal’s requirements. Please let us know if any further modifications are needed.

Sincerely,

Tomoyuki Miyazaki

Attachment Submitted filename: Response to Reviewers.docx

# Peer review: Decision Letter 1

**DOI:** 10.1371/journal.pone.0353835.r003

**Authors:** Yoshitaka Ishibashi

30 Jun 2026

Investigating “Another Me” digital twin technology to support the development of human relationships: An exploratory randomized controlled study

PONE-D-25-59383R1

Dear Dr. Miyazaki,

We’re pleased to inform you that your manuscript has been judged scientifically suitable for publication and will be formally accepted for publication once it meets all outstanding technical requirements.

Within one week, you’ll receive an e-mail detailing the required amendments. When these have been addressed, you’ll receive a formal acceptance letter and your manuscript will be scheduled for publication.

An invoice will be generated when your article is formally accepted. Please note, if your institution has a publishing partnership with PLOS and your article meets the relevant criteria, all or part of your publication costs will be covered. Please make sure your user information is up-to-date by logging into Editorial Manager at Editorial Manager® and clicking the ‘Update My Information' link at the top of the page. For questions related to billing, please contact billing support.

If your institution or institutions have a press office, please notify them about your upcoming paper to help maximize its impact. If they’ll be preparing press materials, please inform our press team as soon as possible -- no later than 48 hours after receiving the formal acceptance. Your manuscript will remain under strict press embargo until 2 pm Eastern Time on the date of publication. For more information, please contact onepress@plos.org.

Kind regards,

Yoshitaka Ishibashi

Academic Editor

PLOS One

Additional Editor Comments (optional):

Dear Dr. Miyazaki Tomoyuki

Thank you for your careful and thorough revision of the manuscript and for your detailed responses. The manuscript now complies with the journal’s requirements. Therefore, I am pleased to recommend that the manuscript be accepted for publication in its current form.

Sincerely,

Yoshitaka Ishibashi

# Editor report: Acceptance letter

**DOI:** 10.1371/journal.pone.0353835.r004

**Authors:** Yoshitaka Ishibashi

PONE-D-25-59383R1

PLOS One

Dear Dr. Miyazaki,

I'm pleased to inform you that your manuscript has been deemed suitable for publication in PLOS One. Congratulations! Your manuscript is now being handed over to our production team.

At this stage, our production department will prepare your paper for publication. This includes ensuring the following:

* All references, tables, and figures are properly cited

* All relevant supporting information is included in the manuscript submission,

* There are no issues that prevent the paper from being properly typeset

You will receive further instructions from the production team, including instructions on how to review your proof when it is ready. Please keep in mind that we are working through a large volume of accepted articles, so please give us a few days to review your paper and let you know the next and final steps.

Lastly, if your institution or institutions have a press office, please let them know about your upcoming paper now to help maximize its impact. If they'll be preparing press materials, please inform our press team within the next 48 hours. Your manuscript will remain under strict press embargo until 2 pm Eastern Time on the date of publication. For more information, please contact onepress@plos.org.

You will receive an invoice from PLOS for your publication fee after your manuscript has reached the completed accept phase. If you receive an email requesting payment before acceptance or for any other service, this may be a phishing scheme. Learn how to identify phishing emails and protect your accounts at https://explore.plos.org/phishing.

If we can help with anything else, please email us at customercare@plos.org.

Thank you for submitting your work to PLOS ONE and supporting open access.

Kind regards,

PLOS ONE Editorial Office Staff

on behalf of

Dr. Yoshitaka Ishibashi

Academic Editor

PLOS One
